# Unraveling the genomic landscape of *Campylorhynchus* wrens along western Ecuador's precipitation gradient: Insights into hybridization, isolation by distance, and isolation by the environment

**DOI:** 10.1002/ece3.11661

**Published:** 2024-07-11

**Authors:** Luis Daniel Montalvo, Rebecca T. Kimball, James D. Austin, Scott K. Robinson

**Affiliations:** ^1^ Florida Museum of Natural History University of Florida Gainesville Florida USA; ^2^ Department of Biology University of Florida Gainesville Florida USA; ^3^ Department of Wildlife Ecology and Conservation University of Florida Gainesville Florida USA

**Keywords:** *Campylorhynchus*, hybridization, isolation by distance, isolation by the environment

## Abstract

Environmental gradients have the potential to influence genetic differentiation among populations ultimately leading to allopatric speciation. However, environmental gradients can also facilitate hybridization between closely related taxa. We investigated a putative hybrid zone in western Ecuador, involving two polytypic wren species (Aves: Troglodytidae), *Campylorhynchus zonatus* and *C. fasciatus*. Our study addressed two primary questions: (1) Is there evidence of population structure and genetic admixture between these taxa in western Ecuador? and (2) What are the relative contributions of isolation by distance and isolation by the environment to the observed genetic differentiation along the environmental gradient in this region? We analyzed 4409 single‐nucleotide polymorphisms (SNPs) from 112 blood samples sequenced using ddRadSeq and a *de novo* assembly. The optimum number of genetic clusters ranged from 2 to 4, aligning with geographic origins, known phylogenetics, and physical or ecological constraints. We observed notable transitions in admixture proportions along the environmental gradient in western Ecuador between *C. z. brevirostris* and the northern and southern genetic clusters of *C. f. pallescens*. Genetic differentiation between the two *C. f. pallescens* populations could be attributed to an unreported potential physical barrier in central western Ecuador, where the proximity of the Andes to the coastline restricts lowland habitats, limiting dispersal and gene flow, especially among dry‐habitat specialists. The observed admixture in *C. f. pallescens* suggests that this subspecies may be a hybrid between *C. z. brevirostris* and *C. fasciatus*, with varying degrees of admixture in western Ecuador and northwestern Peru. We found evidence of isolation by distance, while isolation by the environment was less pronounced but still significant for annual mean precipitation and precipitation seasonality. This study enhances our understanding of avian population genomics in tropical regions.

## Graphical abstract

Environmental gradients influence genetic differentiation and can lead to allopatric speciation or hybridization, as investigated in a hybrid zone involving two wren species in western Ecuador. Our study utilized 4409 SNPs from 112 blood samples, revealing evidence of genetic structure and admixture influenced by geographical and environmental factors. We identified physical barriers that restrict gene flow and observed that isolation by distance plays a more significant role than isolation by environment in shaping genetic differentiation in this region.

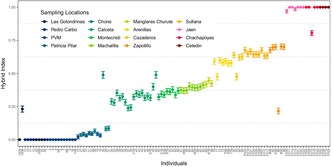

## 1 INTRODUCTION

Geographical barriers are well‐established drivers of diversification in many organisms, including birds and mammals (Tobias et al., 2020; Wright, 1943; Yao et al., 2022). However, other factors, such as hybridization, environmental heterogeneity, and geographical distance can affect the genetic differentiation of populations (DuBay & Witt, 2014; Sexton et al., 2014). These factors remain particularly understudied in tropical species.

The tropics are known to harbor the highest levels of biodiversity, but the true extent of diversity remains underestimated due to incomplete sampling and the lack of detailed studies on many species (Bálint et al., 2011; Lohman et al., 2010). South American bird populations exhibit substantial genetic divergence across geographic space, particularly in species complexes, resulting in high intraspecific differences that overlap with interspecies differences (Brawn et al., 1996; Céspedes‐Arias et al., 2021; de Camargo et al., 2015; Del‐Rio et al., 2022; Milá et al., 2009; Tavares et al., 2011). The Tumbes‐Choco‐Magdalena biodiversity hotspot in South America, which spans a moisture gradient from the humid Choco‐Darien‐Western Ecuador region to the dry Tumbesian region, has a high‐endemism and distinct biogeographical patterns (Dodson & Gentry, 1991; Mittermeier et al., 1998). The presence of endemic species reflects the specialized ecological niches and habitat requirements, contributing to the unique population structures and genetic diversity along hotspots (Hermant et al., 2013).

Environmental heterogeneity promotes the genetic differentiation of populations and facilitates hybridization by providing a pathway for secondary interaction between related species that occur along environmental gradients (Carling & Thomassen, 2012; Randler, 2006; Runemark et al., 2018). Consequently, hybrid zones, regions where genetically distinct populations meet and produce offspring (Harrison & Larson, 2014), commonly occur along environmental gradients (Fritsche & Kaltz, 2000; Kameyama et al., 2008; Yanchukov et al., 2006). Over 200 avian hybrid zones have been formally described, and environmental gradients have been identified as a key factor in maintaining these hybrid zones (Miller et al., 2014). However, the extent of hybridization can vary depending on the strength of reproductive barriers, ecological factors, and genetic compatibility between the species (Ottenburghs, 2018; Winker, 2021). Hybrid zones are fundamental for studying evolutionary processes between divergent populations (Minder et al., 2007; Sloop et al., 2011; Whitham et al., 1999). However, patterns of hybridization and genetic differentiation along environmental gradients in the Tumbes‐Chocó‐Magdalena biodiversity hotspot remain unexplored.

Isolation by distance and isolation by environment are two common models to explain how geographic distance or environmental variability affects the genetic differentiation of populations. Isolation by distance occurs when genetic exchange is inversely proportional to geographic distance (Wright, 1943). Conversely, isolation by the environment is associated with greater genetic similarity between populations in similar environmental conditions (Alberto et al., 2013; Sexton et al., 2014). Research suggests that climatic factors might play a role in driving biogeographical patterns, as closely related species’ distributional boundaries match climate regimen boundaries at the transition zone between the two regions in Western Ecuador (Albuja et al., 2012; Amador et al., 2019; Escribano‐Avila et al., 2017; Morrone, 2006; Prieto‐Torres et al., 2019).

High gene flow between populations from different environments may hinder local adaptation (Tigano & Friesen, 2016). This occurs because gene flow can introduce maladaptive alleles from other populations, disrupting locally adapted genotypes (Tigano & Friesen, 2016). However, moderate gene flow may augment local adaptation by providing genetic variation upon which selection can act (Hoeksema & Forde, 2008). Predicting gene flow patterns across spatial and environmental gradients may facilitate forecasts of species’ resilience to intensifying anthropogenic environmental change (Hoeksema & Forde, 2008). Unfortunately, the effects of hybridization and spatial and ecological processes on genetic differentiation are poorly understood in tropical species.

**FIGURE 1:**
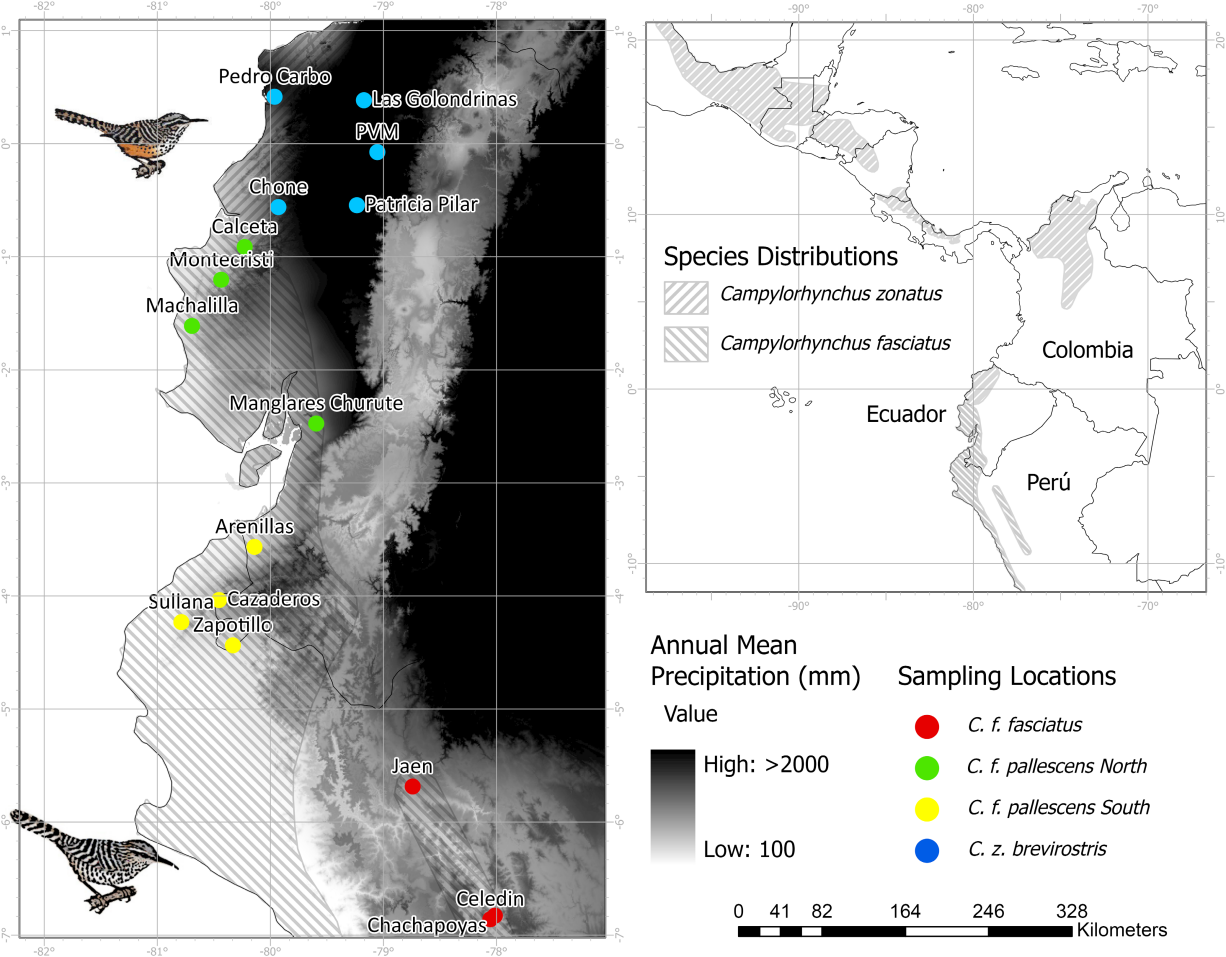
Sampling sites and distribution ranges of *Campylorhynchus zonatus* and *C. fasciatus*. Background colors show the AMP gradient in the region. Black triangle shows the climatic transition and the contact zone of these species.

To address this gap, we investigated patterns of potential hybridization and genetic differentiation of two closely related bird species, the Band‐backed Wren (*Campylorhynchus zonatus brevirostris*) and Fasciated Wren (*Campylorhynchus fasciatus*). The two wren species exhibit parapatric distributions across wet and dry regions of Western Ecuador and Peru, respectively, with the subspecies *C. f. fasciatus* found in the Marañon valley of Northeastern Peru (Figure 1). In addition to the well‐established ecological preferences of *C. zonatus brevirostris* and *C. fasciatus*, field observations of plumage patterns, and frequency suggest that hybridization may occur in the transition zone of the precipitation gradient in western Ecuador (LDM, pers. obs). In the transition zone, some individuals identified as *C. zonatus brevirostris* may lack the ochraceous belly that distinguishes this species (Henry, 2005; Ridgely & Greenfield, 2001), suggesting that they may be potential hybrids of *C. zonatus* and *C. fasciatus*. This suggests that admixed individuals may be present in populations along transitional habitats where *C. zonatus* is locally common. Western Ecuador provides a unique system for studying hybridization and genetic structure patterns in which elevation does not vary. However, the factors underlying genetic differentiation in the region remain unknown.

Given the intermediate phenotypic traits observed in *C. zonatus* and *C. fasciatus* within the transition climatic zone in Western Ecuador, we explored whether there was evidence of genetic admixture and introgression between these taxa, and the patterns of genetic differentiation among these species across environmental gradients in western Ecuador. Moreover, the coincidence of the distribution boundaries of these species with climatic regions prompted us to examine the relative contributions of the isolation by distance and isolation by environment models in shaping the patterns of genetic differentiation along the environmental gradient in Western Ecuador. If ecological conditions and geographical distance influence genetic differentiation and admixture patterns, then we would expect *C. zonatus* and *C. fasciatus* to show significant associations between genetic differentiation, geographical distance, or climate, leading to population structure that closely mirrors the known geographic distribution of species and populations (Brawn et al., 2016; Culumber et al., 2012). Our study elucidates the potential role of hybridization and local adaptation in structuring regional populations. It also discusses the implications of physical barriers for the conservation of dry‐habitat specialists in Southwest Ecuador and explores possible hypotheses to explain introgression patterns among species.

## 2 MATERIALS AND METHODS

### 2.1 Study region

The lowland in Western Ecuador and Peru is characterized by a moisture gradient from the humid Choco‐Darien‐Western Ecuador region in the north to the dry Tumbesian region in the south. Rainfall in Western Ecuador ranges from 2000 to 7000 mm annually, whereas the southern region has an eight‐month dry season with less than 1000 mm annually (Dodson & Gentry, 1991; Figure 1).

### 2.2 Species system

The Band‐backed Wren (*Campylorhynchus zonatus*) is found in eastern Mexico to northwest Ecuador, with seven subspecies and four disjunct populations occupying edges, open disturbed areas, and wet forests (Kroodsma & Brewer, 2020a). One of its subspecies, *C. z. brevirostris*, is present in two disjunct populations in northwestern Ecuador and northern Colombia. The Fasciated Wren (*Campylorhynchus fasciatus*) is a commonly found species in western Ecuador and northwest Peru (Figure 1), where it inhabits arid and semiarid areas and deciduous forests (Kroodsma & Brewer, 2020b; Ridgely & Greenfield, 2001). The subspecies *C. fasciatus pallescens* occurs in western Ecuador and northwestern Peru, while the nominal subspecies *C. f. fasciatus* is present in western Peru and the dry Marañon valley on the eastern side of the Andes (Kroodsma & Brewer, 2020b; Ridgely & Greenfield, 2001). *C. z. brevirostris* and *C. fasciatus* have parapatric distributions along the precipitation gradient in western Ecuador, with *C. z. brevirostris* restricted to the wet region and *C. fasciatus* to the dry region (Ridgely & Greenfield, 2001). Phylogenetic relationships of *Campylorhynchus* show that *C. z. brevirostris* and *C. albobrunneus* are sister species and share a common ancestor with *C. fasciatus* (Barker, 2007; Burleigh et al., 2015; Vázquez‐Miranda & Barker, 2021). Recent studies estimated the time of divergence between *C. z. brevirostris‐albobrunneus* and *C. fasciatus‐pallescens* clades occurred approximately 1.9 million years ago (1.575–2.415 Ma; Vázquez‐Miranda & Barker, 2021). In contrast, the average time to speciation for several phylogroups at the equator is around 2 million years (Weir & Schluter, 2007).

### 2.3 Samples collection

We collected blood samples from the brachial vein of 48 putative *C. z. brevirostris* and 49 *C. f. pallescens* individuals at 12 sampling locations along Western Ecuador, mainly between July and December 2018, with two exceptions in August 2017 (Tables S1 and S2). We stored blood samples in Eppendorf tubes with lysis buffer (Tris HCl, pH = 8.0 = 0.1 M; NaCl = 0.01 M; EDTA = 0.1 M; SDS = 3%). Breeding groups were sampled at least 400 m apart to avoid related individuals. We also obtained tissue samples preserved in 95% ethanol from the Florida Museum of Natural History (FLMNH), including 10 *C. f. pallescens* samples from Sullana in Northwestern Peru (collected in October 2011) and 17 *C. f. fasciatus* samples from three locations in the Marañon valley of northeastern Peru: Jaen (October 2010), Chachapoyas (November 2009), and Celedin (November 2009; Tables S1 and S2). DNA extraction was performed using Qiagen DNeasy blood & tissue kits and Qiagen extraction protocols, with DNA concentration determined using the Qubit dsDNA HS Assay Kit and Qubit® Fluorometer.

### 2.4 Library construction and sequencing

We followed the ddRAD‐seq protocol by Peterson et al. (2012) as modified by Thrasher et al. (2018). Briefly, we digested 20 uL of DNA between concentrations of 3.5–40 ng/uL with Sbfl and Mspl and ligated with one of the 20 P1 adapters (each containing a unique inline barcode) and a P2 adapter (P2‐Mspl). Samples with similar concentrations were pooled in groups of 20 (each with a unique P1 adapter) and purified using 1.5× volumes of homemade MagNA made with Sera‐Mag Magnetic Speed‐beads (FisherSci) as described by Rohland and Reich (2012). Fragments between 400 and 700 bp were selected using BluePippin (Sage Science) by the Cornell University Biotechnology Resource Center (BRC). Index groups and Illumina sequencing adapters were added by performing 11 PCR cycles with Phusion DNA Polymerase (NEB). Multiplexing was performed in several index groups (19 and 20 individuals each), with sequencing on an Illumina NextSeq 500 (150 bp single‐end) incorporating a ~10% PhiX spike‐in for library diversity.

### 2.5 Quality filtering and demultiplexing

After the quality of the reads was assessed using FASTQC version 0.11.5 (Andrews et al., 2016), we trimmed all sequences to 147 bp using fastX_trimmer (FASTX‐Toolkit) to exclude low‐quality calls near the 3′ of the reads. We removed reads containing at least a single base with a Phred quality score of less than 10 (using fastq_quality_filter). We removed sequences if more than 5% of the bases had a Phred quality score of less than 20. Using the process_radtags module from the STACKS version 2.3 (Catchen et al., 2013), we demultiplexed the reads to obtain files with specific sequences for each individual. After demultiplexing, we retained samples with more than 1 × 10^5^ reads for the *de novo* assembly, removing 12 samples with low read numbers ranging from 1.2 × 10^3^ to 9.6 × 10^4^. We ended with a final data set of 112 samples for analysis.

### 2.6 *De novo* assembly

Because we do not have a sequenced genome for any species or a close relative, we assembled the sequences *de novo* using the STACKS pipeline (Catchen et al., 2013). First, we selected 12 samples with the highest number of reads and ran denovo_map.pl testing values from one to nine for ‐*M* (number of mismatches allowed between stacks within individuals) and *n* (number of mismatches permitted between stacks between individuals) following the *n* = *M* rule (Paris et al., 2017) while keeping *m* = 3 (stack depth/number of identical reads required to initiate a new allele). We kept *r* = 0.8 (the minimum percentage of individuals in a population required to process a locus for that population). It has been shown that at least 80% of the population should present a specific locus to be included, known as the 80% rule or r80 loci (Paris et al., 2017). We set all samples to the same population (*p* = 1) for the parameter testing assembly. We plotted the number of single‐nucleotide polymorphisms (SNPs) called against the *M* parameters to find the optimum *M*, after which no additional SNP calling was observed. We found an optimum value for *n* = *m* = 5 for the final *de novo* assembly. After the parameters testing assembly, we performed the *de novo* assembly with all the samples and the parameters described above and set *p* = 1. When a RAD locus had more than one SNP, the data were restricted to the first (‐‐write_single_snp) to avoid including SNPs in high linkage disequilibrium (LD). We required a minor allele frequency of 0.05 to process a nucleotide site (‐‐min_maf).

We used the dartR package (Gruber et al., 2018) to measure pairwise population‐based LD across all loci. We used 0.5 as the threshold for testing SNPs in LD (Carlson et al., 2004). We retained the entire data set for further analyses, given that only 0.1% of loci showed *R* ^2^ values over .5 across all pairwise combinations.

We assessed missing data per individual in our final dataset, ranging from 1% to 38.6%, using VCFtools 0.1.16 (Danecek et al., 2011). To mitigate potential biases in population structure inference via PCA due to missing data, we employed two strategies based on recommendations by Yi and Latch (2022). Firstly, we conducted Spatial PCA (sPCA) analyses, including all samples and excluding nine samples exceeding two standard deviations above the mean missingness (>25% missing data per individual). Additionally, we utilized model‐based methods such as STRUCTURE and DAPC to cross‐validate the PCA results and further elucidate the population structure.

### 2.7 Population structure and admixture patterns

We explored the genetic structure of the data set with a spatial principal component analysis (sPCA) analyzed using the function sPCA from the R package Adegenet 2.13 (Jombart, 2008). We set the function to build a distance‐based connection network with neighbors within a Euclidean distance between 1 and 26.4 km based on the maximum dispersal distance recorded for Cactus Wren (Lynn et al., 2022). The components of the sPCA are separated into global (positive eigenvalues) and local (negative eigenvalues) structures. Large global scores reflect the presence of clines or other structures, while local scores represent the presence of large genetic distances between neighbors. We assessed the significance of both patterns with a Monte Carlo procedure included in the functions global.rtest and local.rtest using 99,999 permutations.

We used the Bayesian clustering software STRUCTURE version 2.3.4; (Pritchard et al., 2000) to estimate the membership coefficients for each individual (*Q*‐value). We ran a spatial (LOCPRIOR = 1) model using sampling locations as prior population information. We used the admixture model running 20 independent replicates per *K*, each with a burn‐in of 10^5^ and a run length of 10^6^ Monte Carlo iterations. We assessed support for genetic clusters (*K*) ranging from 1 to 12, allowing for admixture (NOADMIX = 0; Gilbert et al., 2012). We used the method described by (Evanno et al., 2005) implemented in STRUCTURE HARVESTER version 0.6.94; (Earl & Vonholdt, 2012) to find the value of *K* that captures most of the structure in the data, and that seems biologically sensible (Pritchard et al., 2003). We used the software CLUMPP (Jakobsson & Rosenberg, 2007) with a LargeKGreedy model and 50,000 random repeats to combine replicates accounting for potential “label switching” and “genuine multimodality” differences. We further calculated the posterior probability of assignment of individuals using the discriminant analysis of principal components (DAPC) from the R package Adegenet 2.13 (Jombart, 2008). First, we used the function find.clusters to determine the most likely number of genetic clusters and the group membership for each individual using 100 principal components (PCs) and 10^6^ iterations for *K* = 1–20. We selected the number of clusters with the lowest Bayesian information criterion (BIC) value as optimal. We used the estimated group membership to perform a preliminary DAPC retaining 100 PCs and two discriminant analysis axes (DAs). We used the preliminary DAPC to calculate the optimal number of PCs to keep using the optim.a.score function set with ten simulations. We performed the final DAPCs for *K* = 2–4 using the optimal number of PCs previously estimated.

### 2.8 Parental and hybrid classification

We used the *Q*‐values from STRUCTURE to group individuals as hybrids if 0.1 ≤ *Q*‐value ≤ 0.9 for *K* = 2, and as parental otherwise. We further estimated the Maximum Likelihood of individual hybrid indexes (HI, proportion of alleles inherited from one of the parental species). We classified parental individuals based on whether they belong to the northernmost populations surveyed of *C. z. brevirostris* from Ecuador (Las Golondrinas, Pedro Vicente Maldonado, and Pedro Carbo) or the southernmost allopatric populations surveyed of “nominally” ssp. *C. f. fasciatus* (Jaen, Chachapoyas, and Celedin) and that had *Q*‐values ≥ 0.90 for either parental population, based on STRUCTURE *Q*‐values for *K* = 2. These individuals were designated as “parentals” within the function est.h of the R package INTROGRESS 1.2.3 (Gompert & Alex Buerkle, 2010), which was used to estimate the HI for each individual. The HI ranged from 0 (pure parental *C. z. brevirostris*) to 1 (pure parental *C. f. fasciatus*).

### 2.9 Genetic diversity

We analyzed genetic diversity using the genetic clusters defined by STRUCTURE when *K* = 4. DAPC identified *K* = 4 as the most likely number of clusters, while STRUCTURE regarded it as the third most likely. Employing *K* = 4 not only enables a finer classification but also resulted in genetic clusters with geographical boundaries that correspond closely to those of biogeographical regions that have been previously reported (Amador et al., 2019; Escribano‐Avila et al., 2017; Morrone, 2006; Prieto‐Torres et al., 2019) enabling us to explore potential barriers within Western Ecuador.

We assigned each sample to the genetic clusters if their *Q*‐value for that cluster was greater than 0.9. We estimated alleles frequencies, inbreeding coefficient (*F*_is_) per population, and the observed heterozygosity (Ho) per individual using the function gl.report.heterozygosity from the R package dartR (Gruber et al., 2018). We estimated expected heterozygosity (He) as 2*pq*, where *p* and *q* are the estimated allele frequencies within the genetic clusters. We then calculated the 2.5%, 25%, 50%, 75%, and 97.5% percentiles of observed (Ho) and expected heterozygosity (He) across individuals within these genetic clusters.

We minimized the potential bias of related individuals in genetic diversity estimates (Jankovic et al., 2010) by selecting samples with low kinship coefficients among birds captured in the same mist net. Nei's *F*_ST_ estimates and kinship coefficients were estimated with the HierFstat R package (Goudet, 2005).

We partitioned the total genotypic variance into components due to differences between genetic clusters and differences between individuals within clusters using analysis of molecular variance (AMOVA) with pairwise Nei's *F*_st_ distances between individuals (Nei & Li, 1979). We used the function gl.amova of the dartR package (Gruber et al., 2018) and evaluated significance levels with 9999 permutations. Nei's *F*_st_ (Nei & Li, 1979) provided a more comprehensive understanding of the genetic differentiation among distinct genetic clusters.

### 2.10 Isolation by distance and the environment

We used climate variables from CHELSA 1.2 (Karger et al., 2017). CHELSA is a high‐resolution (30 arc sec, ~ 1 km) free global climate data set. We performed a multiple correlation analysis to identify redundancies among the climatic variables using the Hmisc package for R (Harrell, 2014). We selected the climatic variables that we considered biologically relevant and had the lowest Pearson correlation coefficients with other selected variables to avoid collinearity. After this process, we selected the annual mean temperature, annual mean precipitation, and precipitation seasonality.

We explored patterns of isolation by distance and isolation by the environment using partial Mantel tests, generalized dissimilarity models (GDM), and two distinct dissimilarity datasets. The dissimilarity data sets were based on the mean per sampling location of pairwise Nei's *F*_ST_ (Nei & Li, 1979) and kinship coefficients among samples, normalized as $1-\frac{1-\min\left(x\right)}{\max\left(x\right)-\min\left(x\right)}$, where *x* is the kinship coefficient between two samples. Euclidean distances between coordinates and climate values of each sample were used as predictors of the kinship coefficient matrix. Coordinates and climate values were averaged per sampling location, and then Euclidean distances were estimated and used as predictors for Nei's *F*_ST_ matrix. To account for the Andes as a physical barrier, we conducted the partial Mantel tests and GDM utilizing a dataset that included all sampling sites, and another that excluded the eastern sampling sites (Jaen, Chachapoyas, and Celedin).

First, we correlated both dissimilarity matrices against environmental pairwise Euclidian distances controlled by geography using a partial Mantel set up at 9999 permutations in the R package vegan (Oksanen, 2013). We used the log transformation of environmental and geographic distances—suggested for two‐dimensional habitats—and *F*_st_/(1−*F*_st_) for genetic distances following Rousset (1997). Because the Mantel test tends to inflate type I error (Guillot & Rousset, 2013), we rejected the null hypothesis of no significant correlation if *p*‐value ≤ .001 (Diniz‐Filho et al., 2013). Next, GDM (GDM; Ferrier et al., 2007; Ferrier & Guisan, 2006; Manion, 2009) was used to evaluate the association between genetic dissimilarity datasets as the response variable and environmental and geographic Euclidian distance as predictor variables. This statistical method uses matrix regression to investigate the relationships between dissimilarities in predictor and response variables, and it has been increasingly used in landscape genetic studies (Freedman et al., 2010; Geue et al., 2016; Thomassen et al., 2011). The GDM model combines multiple matrix regressions (I‐splines) into a single non‐linear function to analyze how the dissimilarity between pairs of sampling locations responds to environmental gradients and geographical distance. In particular, the partial regressions of GDM take into account two important factors: (1) the non‐stationary rate of change along an environmental gradient, and (2) the curvilinearity that characterizes the relationship between dissimilarity and environmental gradients (Ferrier et al., 2007; Fitzpatrick et al., 2013). We used the default of three I‐splines per predictor. The significance of the model and predictors was tested with 9999 permutations using the function gdm.varImp of the “gdm” package in R (Fitzpatrick et al., 2022). Significance is estimated using the bootstrapped *p*‐value when the predictor has been permuted. The function also calculates the predictor importance measured as the percent decrease in deviance explained between the full model and the deviance explained by a model fit with that predictor permuted (Fitzpatrick et al., 2022).

### 2.11 Demographic scenarios

We used Momi2 to examine alternative two‐population demographic models that differ in the presence and timing of gene flow between *C. z. brevirostris* and *C. f. pallescens*: (i) pure isolation, (ii) isolation‐with‐migration, and (iii) isolation with secondary contact (bidirectional and unidirectional in either direction). Because Momi2 models gene flow as pulse events, we inserted four equally distant episodes of gene exchange as a function of divergence time for the isolation‐with‐migration model. We kept the effective population sizes (*N*_e_) constant within each model. For the secondary contact model, we constrained the migration events to occur after the most recent time boundary of the Last Glacial Maximum (LGM; ~16,000 years ago; Heine, 2000). We assumed a mutation rate of 4.9 × 10^−9^ substitutions per site per generation (Smeds et al., 2016) and a generation time of 2 years reported for *Campylorhynchus nuchalis* (Bird et al., 2020). We first performed 10 optimizations for each model with ancestral *N*_e_ set to 1 × 10^5^ and the stochastic_optimization function to set 100 mini‐batches and 10 iterations to get initial parameter estimates with reduced computational effort. Based on these results, we used the mean of *N*_e_ (4.7 × 10^5^) and time since divergence (1.63 × 10^5^) across runs and models as initial values for subsequent runs. Here, we report parameter values from models with 50 optimizations and initial values as mentioned above and the function stochastic_optimization set with 1000 SNPs per mini‐batch and 10 iterations. We used the parameter estimations of the 50 runs to generate the mean/median and 95% confidence interval for each estimated demographic parameter. For each model, we selected the optimization with the largest maximum likelihood value for model selection. We used the relative Akaike information criterion to select the best‐fit model (Sakamoto et al., 1986). Finally, we assessed the effect of ancestral *N*_e_ on model selection by running each model twice as described in step two but with 10 optimizations and 10 different ancestral *N*_e_ ranging from 1 × 10^5^ to 1 × 10^6^.

## 3 RESULTS

We obtained 4409 SNPs with an average coverage per individual of 35.4 × (min = 12.8×, max = 77.9×) and an average genotype call rate of 93.5% from 112 individuals and 16 sampling locations.

### 3.1 Population structure and admixture patterns

**FIGURE 2:**
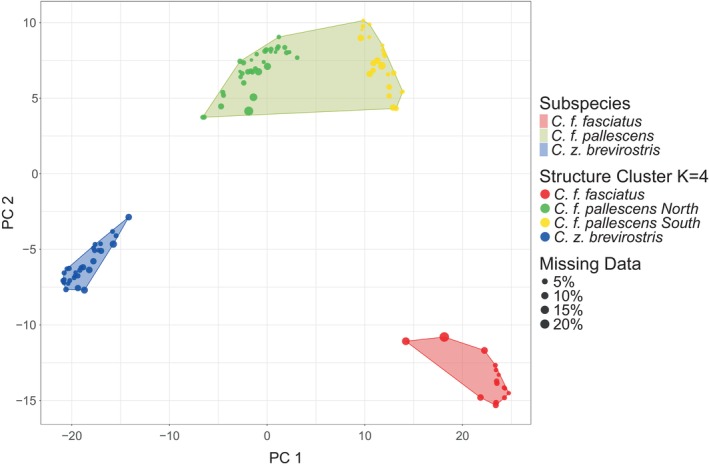
Spatial principal component analysis (sPCA) plot for *Campylorhynchus zonatus* and *C. fasciatus* populations from western Ecuador and northern Peru, derived from ddRADSeq and *de novo* assembly data, excluding samples exhibiting a missing data rate exceeding 25% per individual. Each data point represents a single individual. The polygons delineate groups of individuals according to their subspecies designations based on Ridgely and Greenfield (2001), while the dot colors correspond to the genetic clusters assigned by the STRUCTURE software analysis when *K* = 4. The sPCA plot reveals that the genetic clusters largely align with the subspecies designations, although some degree of overlap on PC1 is observed between *C. f. fasciatus* and *C. f. pallescens* South, suggesting potential gene flow.

The sPCA analysis revealed that the first PC (PC1) accounted for 51.9% of the variance, while the second PC (PC2) explained 16.6%. We found significant evidence for global structure (*p*‐value < .001) but not for local structure (*p*‐value = .905). PC1 divided individuals into four distinct populations corresponding to *C. zonatus*, *C. f. fasciatus*, and two closely related populations of *C. f. pallescens*. The southern populations of *C. f. pallescens* overlapped with *C. f. fasciatus* along PC1, while the northern *C. f. pallescens* populations occupied an intermediate space between the southern population of *C. f. pallescens* and the distinct cluster of *C. zonatus*. PC2 showed three clusters corresponding to *C. zonatus*, *C. f. pallescens*, and *C. f. fasciatus*, with the former two clusters being closer to each other than the latter two (Figure 2). When including samples with more than 25% missing data per individual, the sPCA showed a less clear separation of populations, with some individuals falling into different subspecies than those assigned by model‐based methods, which resulted in broader, overlapping clusters (Figure S1).

**FIGURE 3:**
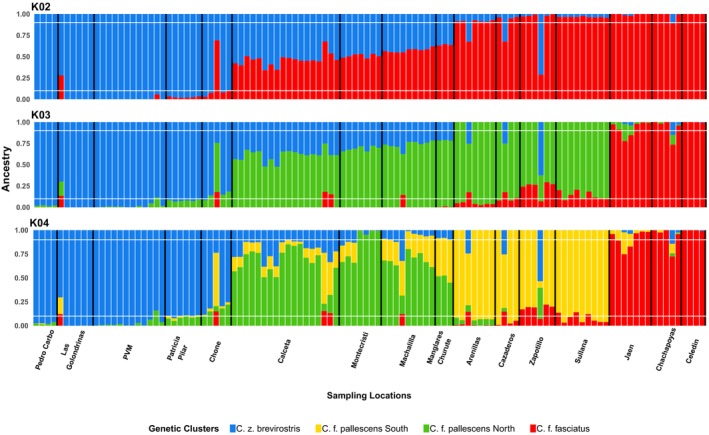
Population structure and admixture patterns in *Campylorhynchus zonatus brevirostris* and *C. fasciatus* along western Ecuador and northern Peru based on ddRADseq data and *de novo* assembly. STRUCTURE results showing individual admixture proportions for *K* = 2–4 genetic clusters. Each vertical bar represents an individual, ordered by sampling location from north to south, with locations separated by black vertical lines. Horizontal white lines indicate ancestry probabilities of 0.1 and 0.9. Distinct genetic clusters correspond to *C. z. brevirostris*, *C. f. pallescens* (North and South), and *C. f. fasciatus*.

STRUCTURE identified *K* = 2 as the most likely number of genetic clusters (Delta *K* = 26,357.04). The second most likely number of genetic clusters was *K* = 3 (Delta *K* = 1880.47), followed by *K* = 4 (Delta *K* = 351.61; Figures 3 and S2). Most of the decrease in Delta values was noted in the transition from *K* = 2 to *K* = 4 clusters. Interestingly, once we surpassed 5 clusters, the Delta value consistently stayed within a range of 170 points, suggesting that the optimal number of clusters for this dataset lies between 2 and 4. We also identified *K* = 2–4 as having the most biological meaning, resembling the clusters observed in the sPCA. Samples from the study region showed increasing admixture from *C. z. brevirostris* from the North to *C. fasciatus* to the south, with admixed individuals falling out in the center (Figure 3). The additional DAPC identified four genetic clusters (Figure S3) as the optimal model (*K* = 4, BIC = 627) and cuts of genetic clusters resembled those from STRUCTURE. Further results from the DAPC showed a sharp decline of BIC for *K* = 2 (641.77) and 3 (630.81).

At *K* = 2, the distribution of assigned groups resembles the geographical boundary previously reported at the species level with *Q* values dropping under 0.9 at the sample sites of Chone and as far as Arenillas. When *K* = 3, the assigned group of *C. fasciatus* on the west slope of the Andes matches the geographical distribution of the subspecies *C. f. pallescens*. The occurrence of admixture between *C. z. brevirostris* and *C. f. pallescens* were predominantly observed in the sampling sites from Chone to Manglares Churute. Hereafter, we refer to this genetic cluster as *C. f. pallescens* North. Admixture between *C. f. pallescens* North and *C. f. fasciatus* was identified in the Southwest region of Ecuador and the Northwest region of Peru. This genetic cluster is hereafter called *C. f. pallescens* South (Figure S4).

**FIGURE 4:**
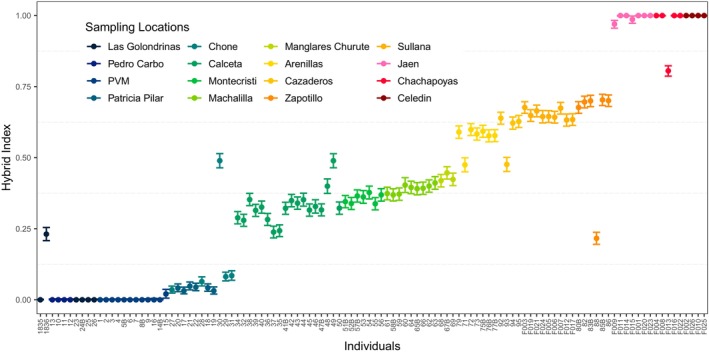
The HI values for *Campylorhynchus zonatus brevirostris* and *C. fasciatus* individuals across their geographic ranges. Each vertical bar represents the HI estimate for an individual, color‐coded by sampling locality. Individuals are ordered latitudinally from northernmost (left) to southernmost (right) sites. Parental *C. z. brevirostris* and *C. fasciatus* individuals have HI values of 0 and 1, respectively. Intermediate values indicate admixed ancestry between the two species. Hybrid indices were estimated using the INTROGRESS R package based on ddRADseq data and the *de novo* assembly.

Hybrid Index (HI) (proportion of alleles inherited from parental *C. f. fasciatus*) estimated by INTROGRESS increased from the northernmost population of *C. z. brevirostris* (HI = 0) to the southernmost population of *C. f. fasciatus* (HI = 1; Figure 4).

Sampling locations associated with the *C. zonatus brevirostris* species exhibited hybrid indices ranging from 0.02 (95% CI 0.006–0.037) at the northernmost non‐parental site of Pedro Vicente Maldonado to 0.085 (95% CI 0.067–0.102) at Chone (Figure 4). Approximately 32.5 km further south, in Calceta, the hybrid index shift to 0.238 (95% CI 0.218–0.259). A progressive increase in the hybrid index was observed southward, reaching 0.447 (95% CI 0.425–0.468) in Manglares Churute, aligning with the genetic cluster of *C. f. pallescens* North (Figure 4). A notable shift in hybrid indices occurred at Arenillas, located approximately 135 km south of Manglares Churute, where the hybrid index rose to 0.590 (95% CI 0.568–0.612), further increasing to 0.703 (95% CI 0.683–0.723) in Zapotillo, falling within the genetic cluster of *C. f. pallescens* South (Figure 4). Most individuals in the eastern Andean region including Jaen, Chachapoyas, and Celedin, were classified as parentals of *C. f. fasciatus*. However, two exceptional samples from Jaen exhibited hybrid indices of 0.970 (95% CI 0.956–0.983) and 0.986 (95% CI 0.973–0.998; Figure 4).

Notably, six samples (30, 88, 49, 93, 71, 1836) exhibited hybrid indices that deviated from the expected geographical range of their assigned genetic cluster. These samples also had a high proportion of missing data per individual, ranging from 30.2% to 32.3%. Nonetheless, the two samples with the highest missing data per individual (60, 48), at 38.6% and 36.8%, respectively, still exhibited hybrid indices within the expected range of *C. f. pallescens* North (Figure 4).

### 3.2 Genetic diversity

The observed heterozygosity decreased whereas the expected heterozygosity increased when moving toward the southern genetic clusters of *C. f. fasciatus*. *C. f. pallescens* North was the only genetic cluster that showed higher observed heterozygosity over expected heterozygosity (Figure S5 and Table S3).

AMOVA showed that 85.4% (*σ* ^2^ = 0.004, *p*‐value < .001) of the genetic variation in our data set was within, while 14.6% (*σ* ^2^ = 0.025, *p*‐value < .001) was among genetic clusters (Table S4). Nei's *F*_ST_ showed that the genetic differentiation among distinct genetic clusters was primarily attributed to the differentiation between *C. z. brevirostris* and other genetic clusters. *C. z. brevirostris* showed the highest Nei's *F*_ST_ value (0.088) when compared to *C. f. pallescens* South, and the lowest (0.058) when compared to *C. f. fasciatus* (Table S5).

### 3.3 Isolation by distance and the environment

Across the sampling sites, the average annual mean temperature (AMT) exhibited relatively low variation, ranging from approximately 21–25°C. Notably, slightly higher temperatures were observed at a few specific locations, including Pedro Carbo and Manglares Churute (Table S6). Annual precipitation displayed substantial heterogeneity. Notably, for *C. z. brevirostris*, sites like Las Golondrinas and Pedro Vicente Maldonado received very high annual rainfall, exceeding 2500 mm. Conversely, *C. f. pallescens* South populations at locations such as Sullana experienced significantly lower yearly precipitation, with values around 200 mm (Table S6). Precipitation seasonality (PS) mirrored the pattern observed for annual mean precipitation (AMP). Habitats supporting *C. f. fasciatus*, particularly those at Jaen and Celedin, exhibited low seasonality, with values ranging from approximately 32–47. Conversely, habitats for *C. f. pallescens* South, especially Zapotillo and Sullana, displayed extreme seasonality, exceeding 140 (Table S6).

Geographical distance was the main factor explaining genetic variation across all analyses, regardless of whether the eastern Andes sampling sites were included, the genetic distance metric used (Nei's *F*_ST_ or the kinship coefficient), or the statistical method (Mantel test or GDM; Tables 1 and 2). The only exception was the Mantel test using Nei's *F*_ST_, which did not reach significance at the *α* level of .001 (*r* = .638, *p*‐value = .0013; Table 1).

**TABLE 1:** Mantel tests were used to evaluate the effects of geographic distance and environmental factors on genetic differentiation in *Campylorhynchus* wrens in western Ecuador and northern Perú.

Mantel tests	Geographic	AMT	AMP	PS	All predictors
Nei's *F*_ST_					
Model with all sites	0.823 (<0.001)	0.237 (0.07)	0.380 (0.02)	0.203 (0.097)	0.363 (<0.05)
Model only west sites	0.661 (<0.05)	0.146 (0.105)	0.661 (<0.001)	0.246 (<0.05)	0.609 (<0.001)
Kinship coefficients					
Model with all sites	0.755 (<0.001)	−0.312 (1)	0.540 (<0.001)	0.013 (0.332)	0.539 (<0.001)
Model only west sites	0.581 (<0.001)	−0.044 (0.932)	0.403 (<0.001)	0.157 (<0.001)	0.403 (<0.001)

*Note*: Genetic dissimilarity was quantified using Nei's *F*_ST_ among sampling sites and kinship coefficients among individuals derived from ddRADseq data. Analyses were performed both including all sampling sites and excluding sites east of the Andes Mountains. Mantel tests assess the correlation between genetic and predictor distance matrices. Environmental predictors are the AMT, AMP, and PS. Significant Mantel correlations indicate stronger effects on genetic differentiation.

Abbreviations: AMP, annual mean precipitation; AMT, annual mean temperature; PS, precipitation seasonality.

**TABLE 2:** GDMs were used to evaluate the effects of geographic distance and environmental factors on genetic differentiation in *Campylorhynchus* wrens in western Ecuador and northern Perú.

GDMs	Predictor importance				Model assessment	
	Geographic	AMT	AMP	PS	Model deviance	Percentage deviance explained
Full Model						
Nei's *F*_ST_						
Model with all sites	24.178 (<0.001	0.772 (0.275)	5.956 (<0.05)	3.705 (0.038)	1.730	87.94 (<0.001)
Model only west sites	22.314 (<0.001	1.007 (0.372)	10.519 (<0.05)	1.655 (0.233)	1.149	81.426 (<0.001)
Kinship coefficients						
Model with all sites	24.045 (<0.001)	0.233 (0.757)	39.924 (<0.001)	0 (NA)	244.291	51.598 (<0.001)
Model only west sites	13.374 (<0.001)	0 (NA)	14.455 (<0.05)	1.154 (0.364)	150.446	45.098 (<0.001)

*Note*: Genetic dissimilarity was quantified using Nei's *F*_ST_ among sampling sites and kinship coefficients among individuals derived from ddRADseq data. Analyses were performed both including all sampling sites and excluding sites east of the Andes Mountains. GDM results show the model deviance, percentage of deviance explained by the full model, and predictor importance when permuting each variable (bootstrapped *p*‐values). Environmental predictors are the AMT, AMP, and PS. Higher deviance explained indicate stronger effects on genetic differentiation.

Abbreviations: AMP, annual mean precipitation; AMT, annual mean temperature; GDMs, generalized dissimilarity models; PS, precipitation seasonality.

The AMP was a significant predictor of genetic variation in all models using kinship coefficients, both with and without the eastern Andes sampling sites. It was also significant in the GDM using Nei's *F*_ST_ distances, but only when the eastern Andes sampling sites were excluded (predictor importance = 9.088, *p*‐value < .05; Table 1). The PS was only significant in predicting kinship coefficients in the Mantel test without the eastern Andes sampling sites (*r* = .157, *p*‐value < .001). The AMT was insignificant in any models (Tables 1 and 2).

### 3.4 Demographic scenarios

The best‐fit demographic model was isolation with secondary contact and asymmetric gene flow from *C. z. brevirostris* toward *C. f. pallescens* North (For the ancestral *N*_e_ = 4.7 × 10^5^, AIC = 23,800.64; Table 3, Figure S6). This model generally had the largest relative likelihood and lowest AIC across the two replicates and ten different ancestral *N*_e_, except for one replicate with ancestral *N*_e_ of 2 × 10^5^, 3 × 10^5^, 7 × 10^5^ where three different models were selected. *N*_e_ at the time of divergence in the isolation model with secondary contact and North–South gene flow was 2.77 × 10^6^ individuals (95% CI 2.77 × 10^6^–2.79 × 10^6^) for *C. z. brevirostris* and 6.87 × 10^4^ (95% CI 5.90 × 10^4^–6.96 × 10^4^) for *C. f. pallescens* North. Gene flow from the north to the south was 33.38% of *N*_e_ (95% CI 30.01%–44.21%). *C. z. brevirostris* and *C. f. pallescens* diverged around 0.93 million years ago (Ma; 95% CI 8.48 × 10^4^–1 × 10^5^ years; Table S7). The best‐fitting model suggests that this migration event occurred 9.41 × 10^3^ years ago (95% CI 8.13 × 10^3^–1.07 × 10^4^ years; Table S7).

**TABLE 3:** Model selection results from Momi2 evaluating demographic scenarios of divergence with gene flow between the lineages *Campylorhynchus zonatus brevirostris* and *C. f. pallescens* based on ddRADseq data.

Demographic scenario	Migration direction	AIC	Delta AIC	AIC weight
Isolation without migration	No migration	24,483	682.21	1.18E‐58
Isolation with migration	Bidirectional	24,043	187.92	1.56E‐41
Secondary contact	Bidirectional	23,844	43.6	3.41E‐10
	North–South*	23,801	0	1
	South–North	24,004	202.87	8.85E‐45

*Note*: The Table shows the demographic model scenario, the direction of modeled gene flow, AIC value, delta AIC relative to the best‐fit model, and AIC weight for each model. Lower AIC and delta AIC values indicate better fit to the genomic data. The highest AIC weight (*) corresponds to the best‐supported isolation model with asymmetric introgression from *C. zonatus* into *C. f. pallescens*.

## 4 DISCUSSION

Our study revealed evidence of genetic structure in the populations of *Campylorhynchus* wrens in western Ecuador and northern Peru. The identified genetic clusters exhibited boundaries corresponding to the species replacement of *Campylorhynchus zonatus brevirostris* and *Campylorhynchus fasciatus pallescens*, and physical barriers to dispersal. The most likely number of genetic clusters ranged from 2 to 4, corresponding to categories defined by geographic origins, estimated phylogenies, and known physical or ecological constraints. We observed admixture patterns, with notable transitions in admixture proportions along the environmental gradient in western Ecuador between *C. z. brevirostris* and the northern genetic cluster of *C. f. pallescens*, extending further south to the southern cluster of *C. f. pallescens*. Furthermore, our analyses provided evidence for isolation by the environment, influenced by the AMP, and isolation by distance across statistical analyses and datasets.

### 4.1 Population structure and admixture patterns

Although differentiation between western and eastern populations of *C. fasciatus* was expected due to the Andes as a barrier to gene flow, the differentiation of the two western populations of *C. f. pallescens* was not expected. Sampling sites assigned to *C. f. pallescens* in Southwest Ecuador and Northwest Peru formed a discrete group distinct from those in Midwest Ecuador. Heterozygosity patterns coupled with the cline of the ancestry probabilities from STRUCTURE (Figure 3), DAPC (Figure S3), and hybrid index (Figure 4) suggested hybridization between *C. f. pallescens* and *C. z. brevirostris* in the contact zone in western Ecuador (Chone).

We believe that admixture events between *C. z. brevisrostris* and *C. f. pallescens* North are suggested to have played a role in the much higher Ho estimated for *C. f. pallescens* North than found in other clusters (Figure S5, Table S3). The contact zone between hybridizing taxa is expected to exhibit higher levels of heterozygosity (Boca et al., 2020). We hypothesize that reduced gene flow across the Andes may have contributed to the low Ho in *C. f. fasciatus*. Nonetheless, it is essential to note that small sample sizes may inflate heterozygosity levels (Schmidt et al., 2021), though utilizing appropriate estimators and a substantial number of bi‐allelic markers (>1000), it may be possible to use as few as four individuals (Willing et al., 2012). Thus, it is noteworthy that *C. f. fasciatus* had the lowest sample size (*n* = 12) and observed heterozygosity values (Table S3).

Seven samples (30, 49, 60, 71, 88, 93, 1836) emerged as outliers exhibiting heterozygosity exceeding 30% (Figure S5). As previously noted, six of these outlier samples also exhibited high hybrid index values and a substantial percentage of missing data per individual. Five samples with high missing data belonged to the *C. f. pallescens* South genetic cluster. Despite the presence of these outlier samples, the observed heterozygosity within the *C. f. pallescens* South cluster remained lower than the expected heterozygosity (Figure S5). While these outliers could potentially diminish the disparity between observed and expected heterozygosity within this cluster, the prevailing trend of lower observed heterozygosity compared to the expected levels was maintained.

Furthermore, differences in heterozygosity between *C. z. brevirostris* and *C. f. fasciatus* could also be explained by a Wahlund effect, characterized by a decrease in heterozygosity due to a fine‐scale population subdivision not accounted for in the sampling (Freeland et al., 2011). Nonetheless, no strong evidence was found to support further population subdivision in our population structure analyses that could lead to the Wahlund effect.

Incomplete lineage sorting (ILS) can also generate genetic diversity patterns comparable to those caused by hybridization (Huerta‐Sánchez et al., 2014). ILS refers to the retention and stochastic sorting of ancestral polymorphisms (Maddison et al., 2006). ILS and secondary gene flow can be distinguished when geographic distribution information is available by comparing patterns of genetic diversity between pairs of neighboring and distantly located populations of the different species (Muir & Schlötterer, 2005). Gene flow is expected to occur preferentially between neighboring populations, resulting in higher intraspecific genetic diversity and lower interspecific genetic differentiation than between distantly located populations (Petit & Excoffier, 2009). In contrast, shared polymorphisms are expected to be distributed evenly across all populations under the ILS scenario (Petit & Excoffier, 2009; Zhou et al., 2017). In this study, the increase in the frequencies of alleles as shown by the hybrid index (proportion of alleles inherited from parental *C. fasciatus*) differs from the expected allele frequency pattern randomly distributed across two species in ILS. Furthermore, the coalescent‐based demographic analysis would identify isolation‐with‐migration as the best‐fit model under ILS (Wang et al., 2019). In contrast, the best model was isolation with secondary contact and asymmetrical gene flow (Table 3).

### 4.2 Manglares Churute as a barrier to gene flow

The lowlands (from 0 to 800 m above the sea level) between the Andes and the coastline constitute a 16 km wide corridor near Manglares Churute (a.k.a. Maglares Churute Corridor, MCC). Habitats in the Manglares Churute corridor are discontinuous and consist primarily of lentic bodies of water, wetland, second‐growth, and evergreen forests with few deciduous and semideciduous remnants (Alava et al., 2007; BirdLife International, 2022). In such geographical settings, dispersal between adjacent sites in a one‐dimensional stepping‐stone model may be limited (Kimura & Weiss, 1964). Higher precipitation of the Andean slopes breaks the continuity of arid habitats along the Manglares Churute corridor, so dispersal and gene flow may be more difficult for dry‐habitat specialists in this region. The effect of isolation by distance restricting gene flow is intensified along narrow corridors, particularly for short‐range dispersal species (Kimura & Weiss, 1964; Wright, 1943). Typical dispersal distances for most Troglodytidae remain poorly understood. Current knowledge indicates that Cactus Wrens, for instance, can disperse up to a maximum distance of 26 km and an average of 2 km (Lynn et al., 2022). Additionally, it is known that cooperative breeding systems may impose constraints on dispersal (Hatchwell, 2009). We hypothesize that the *Campylorhynchus* dispersal characteristics and environmental and geographical factors likely contribute to the restricted gene flow along the Manglares Churute corridor.

The barrier to gene flow that the Manglares Churute corridor imposes on terrestrial lowland species—coupled with anthropogenic threats—might have significant consequences for conservation and evolution (Wagner & Fortin, 2013). Other species that show morphometric and plumage differentiation across the Manglares Churute corridor could have similar genetic patterns. For example, Necklaced Spinetail (*Synallaxis stictothorax*) has two races: the nominal *stictothorax* occurs north of Manglares Churute corridor whereas *maculata* occurs south of the Manglares Churute corridor (Ridgely & Greenfield, 2001). The same pattern is observed for the nominal race of Collared Antshrike (*Thamnophilus bernardi*), which occurs north of Manglares Churute corridor. In contrast, *piurae* occurs south of the Manglares Churute corridor (Ridgely & Greenfield, 2001). We suspect species such as Blackish‐headed Spinetail (*Synallaxis tithys*) might exhibit similar genetic differentiation across Manglares Churute corridor. If this is correct, it would mean that cryptic biodiversity in the dry forest of west‐central Ecuador might need additional conservation attention. We propose that Manglares Churute corridor may be an essential barrier to gene flow for lowland dry‐habitat specialists and should be explored in future studies.

### 4.3 Isolation by distance and the environment

The sampling sites of Pedro Carbo and Manglares Churute, situated at the climatic and geographic peripheries of their respective genetic clusters (Figures 1 and S4, Table S6), exhibited lower sample sizes, with only four and three *Campylorhynchus* individuals captured at each site, respectively (Table S2). Field observations corroborated the reduced species abundance at these locations, potentially attributable to geographical isolation—Pedro Carbo by the coastline and Manglares Churute within a restricted corridor. Sites positioned at the edges of a genetic cluster's climatic and geographic distribution may encounter environmental constraints that limit species abundance (Fristoe et al., 2023; Martin et al., 2024). This phenomenon aligns with the concept of ecological marginality, which posits that populations inhabiting the periphery of their species' range often experience suboptimal conditions, leading to decreased population density and abundance (Pironon et al., 2017; Sexton et al., 2009). In contrast, Calceta and Chone, located within a transitional zone and on the climatic periphery, are not subject to substantial geographic barriers and exhibited higher abundance. This observation suggests that geographic accessibility may attenuate the effects of climatic marginality, enabling gene flow or dispersal from less isolated areas, thereby sustaining higher abundance. The role of gene flow in mitigating the impacts of environmental marginality has been documented in various taxa (Alleaume‐Benharira et al., 2006; Kawecki, 2008). Furthermore, the ability of species to disperse from core populations to marginal areas has been shown to contribute to the persistence of peripheral populations (Channell & Lomolino, 2000; Holt, 2003). However, the contrast in abundance between these sites warrants further investigation.

We found potential evidence for isolation by distance and isolation by the environment, supported by the significant positive relationship between geographic and AMP and genetic distances. The extent, pattern, and consistency of gene exchange in transitional zones can be explained by both environment‐independent (endogenous) and environment‐dependent (exogenous) selection (exogenous; Pyron & Burbrink, 2013). Thus, if the selection is exogenous, a clinal genetic pattern like the one reported in this study (Figures 3 and 4) may be maintained through differential selection across an environmental gradient, such as a climatic boundary (Haldane, 1948; Harrison, 2012).

It is essential to recognize that although patterns of isolation by environment help identify potential systems for adaptive divergence (Wang & Bradburd, 2014), evidence for isolation by environment does not necessarily imply that local adaptations are involved. Isolation by the environment can arise from several mechanisms other than selection and can be confounded with incipient ecological speciation (Wang & Bradburd, 2014). Discerning the relative contribution of geography and environment in shaping genetic diversity remains challenging (Saenz‐Agudelo et al., 2015). One major complication in discriminating between these two factors in evolution is that geographical distance and environmental differences are often correlated (Saenz‐Agudelo et al., 2015; Wang & Bradburd, 2014). Although efforts were made to account for collinearity among predictors in the statistical analyses, it cannot be completely ruled out that collinearity may have affected the reported association in this study.

### 4.4 Demographic scenarios and mechanisms of introgression

The increasing admixture (Figures 3 and 4) and the best‐fit demographic model (Table 3) suggest introgression from *C. z. brevirostris* into *C. f. pallescens* North. The second best‐fit model was isolation with secondary contact and south–north gene flow, implying that gene flow in the opposite direction may also be possible (Table 3). As far as we know, no hybridization involving *C. fasciatus* has been reported previously, but hybridization between *C. albobrunneus* (White‐headed Wren) of western Colombia and Panama and *C. z. brevirostris* of Ecuador has been suggested in the north of Ecuador (Ridgely & Greenfield, 2001).

One driver of asymmetrical introgression, as we observed here, can be caused by sexual selection through a combination of female choice or male–male interactions (Martin & Mendelson, 2016; Stein & Uy, 2006). In some circumstances, the direction of the introgression is likely to be driven by the sex that determines reproductive choices. For instance, heterospecific female pairing preference for the aggressive golden‐collared males in a manakin hybrid zone caused asymmetric introgression of plumage traits into the less aggressive white‐collared manakin (Stein & Uy, 2006). The lack of female preference for either hetero or conspecifics could produce similar patterns. For example, introgression skewed toward the Small Tree‐Finch (*Camarhynchus parvulus*) in the Galápagos Archipelago was associated with the lack of assortative preference of females of the rarer Medium Tree‐Finch (*Camarhynchus pauper*; Peters et al., 2017). Data on female mating preferences are needed to determine whether female choice drives the introgression of *C. z. brevirostris* into *C. f. pallescens* North.

Interspecific territoriality—a common type of interference competition in animals—is strongly associated with bird hybridization, implying that reproductive interference favors the maintenance of interspecific territoriality (Cowen et al., 2020; Drury et al., 2020). Interspecific territoriality leads to confrontation between competitors. As a result—regardless of their foraging efficiency—losers in these interactions are frequently excluded from all resources defended by the dominant individual (Grether et al., 2013; Gröning & Hochkirch, 2008). Interspecific territoriality could be driving the introgression of *C. z. brevirostris* toward *C. f. pallescens* North if the former is the dominant species. Introgression driven by a dominant species is a pattern found in other species (McDonald et al., 2001; Pearson & Rohwer, 2000). Aggressive interference studies are needed to understand the dominant behavior between these species, and whether it is consistent with the directionality of introgression. While the underlying mechanisms behind male–male interactions and female choice differ, they are not mutually exclusive.

When hybrid fitness depends on ecological conditions, fitness consequences of hybridization may vary with environments or fitness components (Harrison, 2012; Parris, 2001). In such cases, alleles conferring greater fitness under specific ecological conditions may determine the direction of introgression (Coster et al., 2018). Ecological factors might be influencing the introgression of *C. z. brevirostris* into *C. f. pallescens* North if the former has alleles adapted to the climate along the transition zone. An experimental approach to study physiological adaptations or genome‐environment association studies could help unravel climate as a driving force for introgression between these two species.

The direction of introgression may be influenced by demographic scenarios, such as the highly different population sizes of hybridizing species (Currat et al., 2008; Lepais et al., 2009). According to the “Hubbs principle”—also known as the “desperation theory”— (Hubbs, 1955; Randler, 2002), birds are more prone to hybridize when the number of individuals in one or both species is limited (Currat et al., 2008; Lepais et al., 2009). The Hubbs principle would predict introgression from *C. f. pallescens* North into *C. z. brevirostris*. In contrast, our results showed that gene flow tends to move toward the larger and more abundant species *C. f. pallescens* North.

The estimated time of migration of the best model (9.41 × 10^3^ years ago, 95% CI 8.13 × 10^3^–1.07 × 10^4^ years) coincides with approximately 5.5 × 10^3^ years after the end of the Last Glacial Maximum, dated to have concluded around 1.6 × 10^4^ years ago (Heine, 2000). The Last Glacial Maximum was marked by a significant shift to cooler, and drier climatic conditions (Heine, 2000). The timing of this secondary contact event suggests that the populations likely came into contact during a period of relatively warmer and more favorable climatic conditions following the conclusion of the Last Glacial Maximum, potentially facilitating gene flow and admixture between the previously isolated populations.

While we accounted for potential biases introduced by ancestral population changes by running each demographic model with 10 different ancestral population sizes, maintaining constant daughter population sizes could potentially bias the outcomes towards secondary contact scenarios (Momigliano et al., 2021). However, our conclusions on admixture between *C. zonatus brevirostris* and *C. f. pallescens* are further supported by the STRUCTURE and DAPC analyses, as well as the estimated hybrid indices. Furthermore, the conclusion of introgression toward the *C. f. pallescens* North genetic cluster is supported by the higher observed heterozygosity relative to the expected heterozygosity in this cluster, coupled with the lower admixture levels observed in *C. zonatus brevirostris*. Collectively, these findings suggest a pattern of introgression from *C. z. brevirostris* into the *C. f. pallescens* North population.

Certain models exhibited notably high AIC values for specific replicates and ancestral effective population sizes (Figure S6). This phenomenon could potentially be attributed to pathological runaway behavior, a common issue in SFS‐based demographic inference algorithms, wherein inferred population sizes and epoch durations may either degenerate to zero or diverge to infinity (Rosen et al., 2018). Such behavior can result in unstable and biased estimates of parameters, impacting not only population sizes and epoch durations but also migration rates and divergence times (Terhorst & Song, 2015). Consequently, these parameters should be interpreted with caution.

### 4.5 Hybridization and conservation in western Ecuador

Conserving tropical bird diversity requires a better understanding of hybridization, particularly in the face of climate change and habitat loss (Radley et al., 2021). While traditionally viewed as a threat to genetic integrity (Casas et al., 2012), hybridization can potentially act as a buffer against climate change by facilitating the transfer of adaptive traits between species (Brauer et al., 2023; Vedder et al., 2022). This highlights the need for conservation strategies that consider both hybridization's risks and potential benefits.

The Tumbes–Choco–Magdalena biodiversity hotspot in western Ecuador exemplifies this challenge. Climate change projections predict increased rainfall in drier southern regions (Marengo et al., 2012), further exacerbating the effects of habitat loss on birds and mammals (Mantyka‐Pringle et al., 2015). Predicted changes, including heat waves (McKechnie & Wolf, 2010) and prolonged dry seasons (Brawn et al., 2016), could lead up to 43% range contractions (Velásquez‐Tibatá et al., 2013).

Populations at niche margins, such as those around the sampling sites of Chone and Manglares Churute, where species ranges approach current and future climate niche limits, likely hold genetic diversity critical for adaptation to changing climate (Singhal et al., 2021). By overlaying genetic monitoring efforts with areas of niche marginality, we can identify where genetic monitoring coincides with anticipated climate change effects on biodiversity (Singhal et al., 2021).

Understanding the impact of climate on genetic diversity is essential for effective conservation strategies in the face of climate change. By characterizing current ranges and assessing whether species harbor and exchange adaptive genetic variants, we can predict their responses to future climates and inform conservation strategies for wrens and other species with similar distributional patterns.

## AUTHOR CONTRIBUTIONS

**Luis Daniel Montalvo:** Conceptualization (lead); data curation (lead); formal analysis (lead); investigation (lead); methodology (lead); project administration (lead); visualization (equal); writing – original draft (equal); writing – review and editing (equal). **Rebecca T. Kimball:** Conceptualization (equal); investigation (equal); methodology (equal); supervision (equal); validation (equal); writing – review and editing (equal). **James D. Austin:** Investigation (supporting); methodology (supporting); supervision (supporting); validation (supporting); writing – review and editing (supporting). **Scott K. Robinson:** Conceptualization (equal); funding acquisition (equal); investigation (equal); methodology (equal); supervision (equal); validation (equal); writing – review and editing (equal).

## CONFLICT OF INTEREST STATEMENT

The authors state that there are no conflicts of interest that might affect the research presented in this paper.

## Supporting information


Data S1.

## Data Availability

All sequencing data from this study have been deposited at NCBI Sequence Read Archive under Bioproject accession number PRJNA925654 at https://submit.ncbi.nlm.nih.gov/subs/sra/SUB12536456/overview. All bioinformatic pipelines, sequence alignments and analytical scripts are available on GitHub https://github.com/ldmontalvo/Landscape‐Genomic‐Wrens.
