# Supplementary material for: Unraveling the genomic landscape of Campylorhynchus wrens along western Ecuador's precipitation gradient: Insights into hybridization, isolation by distance, and isolation by the environment
Source: Ecol Evol. 2024 Jul 11;14(7):e11661. doi: 10.1002/ece3.11661 (PMC11237350; doi:10.1002/ece3.11661)
Supplement: Supplementary file 1 — Data S1. [file ECE3-14-e11661-s001.docx]

**Supplementary Files**

**Content: Supplementary figures and tables for Montalvo et al. “Unraveling the Genomic Landscape of *Campylorhynchus* Wrens along Western Ecuador's Precipitation Gradient: Insights into Hybridization, Isolation by Distance, and Isolation by Environment”**


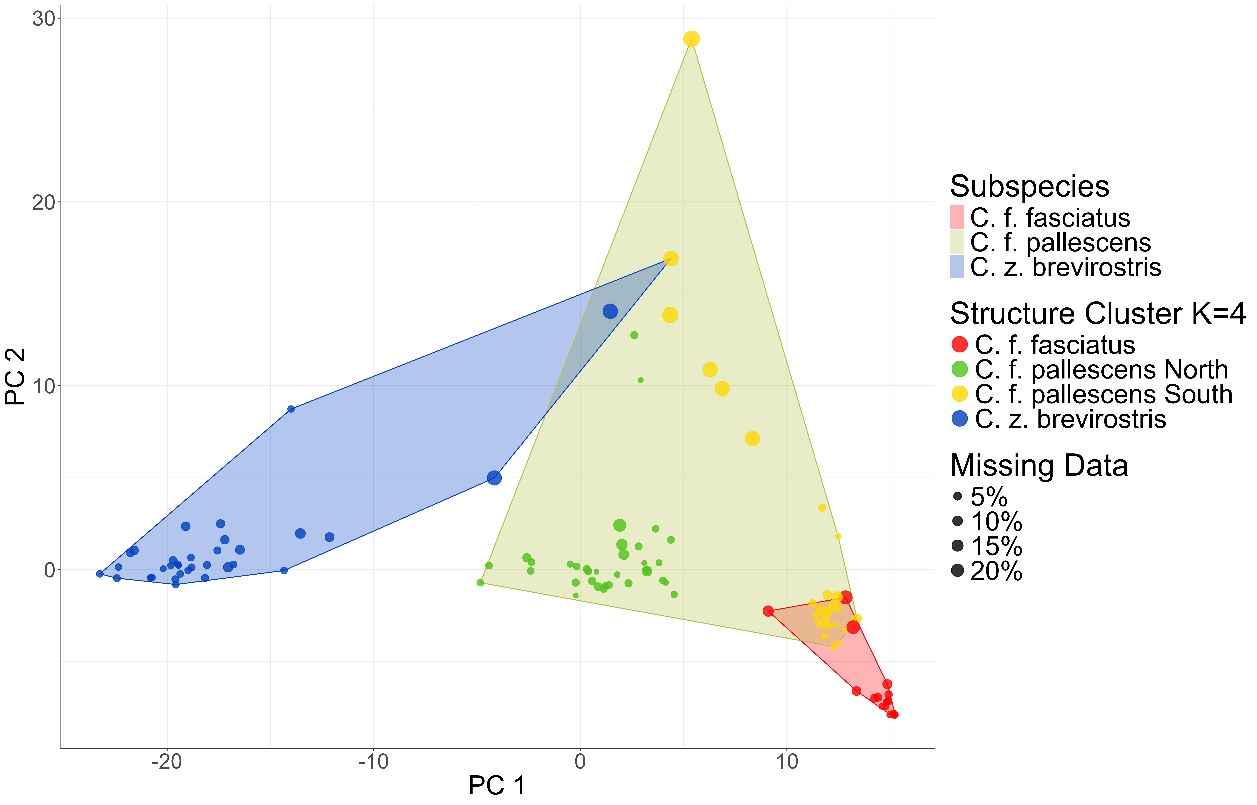


Figure S1. Spatial Principal Component Analysis (sPCA) plot for *Campylorhynchus zonatus* and *C. fasciatus* populations from western Ecuador and northern Peru, derived from ddRADSeq and de novo assembly data, including samples exhibiting a missing data rate exceeding 25% per individual. Each data point represents a single individual. The polygons delineate groups of individuals according to their subspecies designations based on Ridgely and Greenfield (2001), while the dot colors correspond to the genetic clusters assigned by the STRUCTURE software analysis when K=4. The sPCA plot reveals that the genetic clusters largely align with the subspecies designations, although some degree of overlap is observed, primarily attributed to the inclusion of samples with more than 25% missing data per individual.


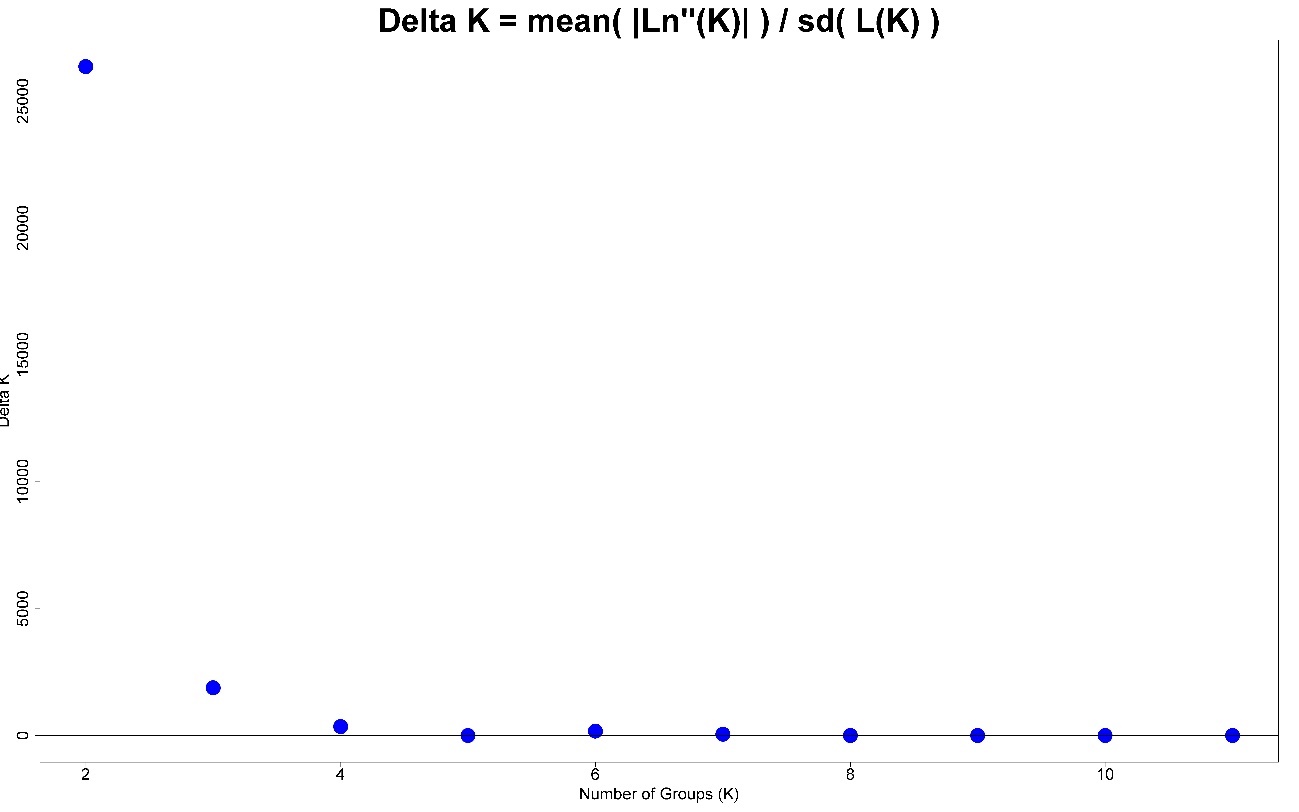


Figure S2. Delta K values for each K as estimated by the software STRUCTURE, do not show significant decreases when samples are split into more than four groups, indicating the optimum number of populations for *Campylorhynchus zonatus* and *C. fasciatus* along western Ecuador and northern Peru based on ddRADseq data and de novo assembly.


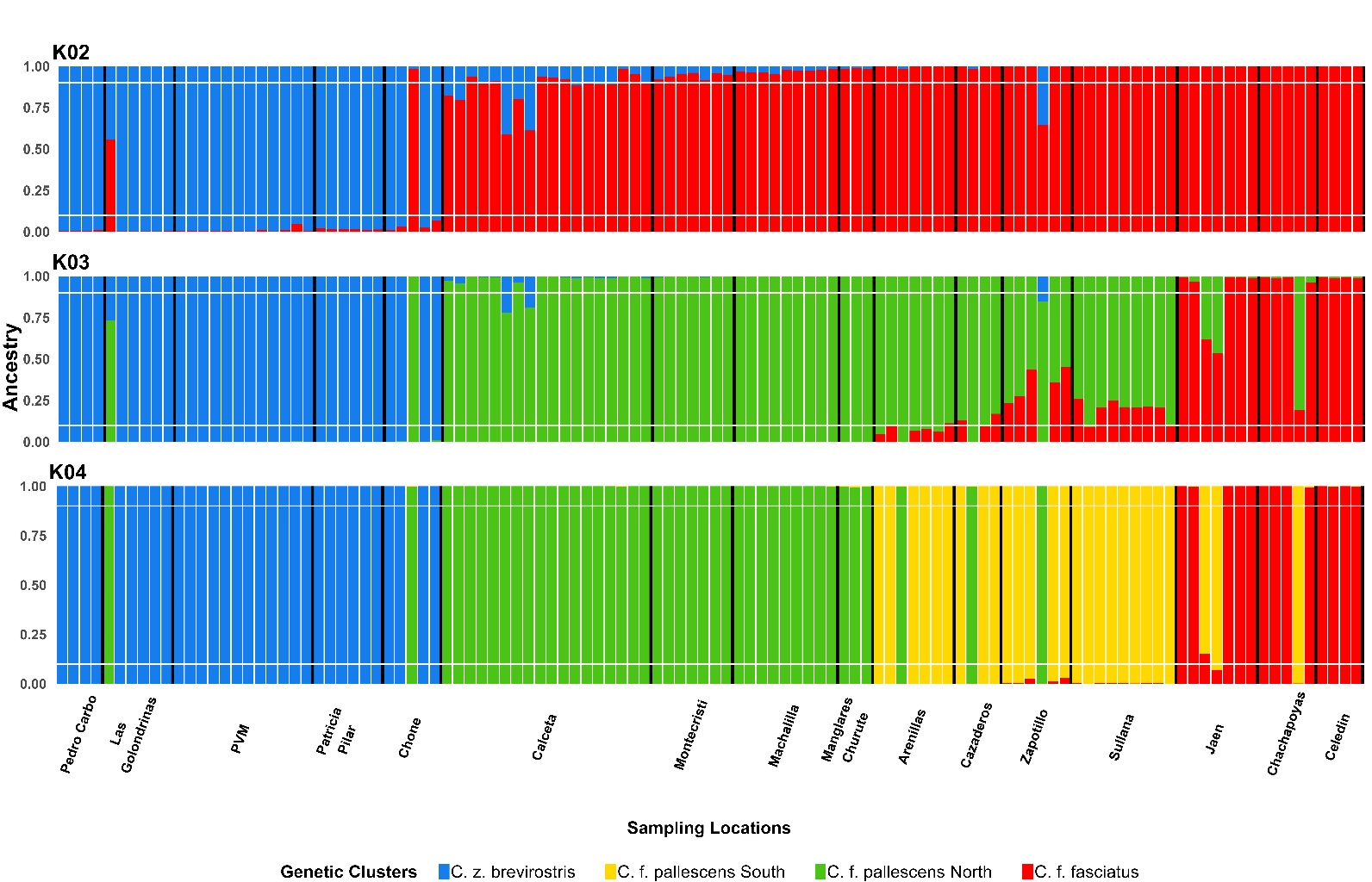


Figure S3. Population structure and admixture patterns in *Campylorhynchus zonatus* and *C. fasciatus* along western Ecuador and northern Peru based on ddRADseq data and de novo assembly. Discriminant Analysis of Principal Components (DAPC) results showing individual admixture proportions for K = 2-4 genetic clusters. Each vertical bar represents an individual, ordered by sampling location from north to south, with locations separated by black vertical lines. Horizontal white lines indicate ancestry probabilities of 0.1 and 0.9. Distinct genetic clusters correspond to *C. z. brevirostris, C. f. pallescens* (North and South), and *C. f. fasciatus*.


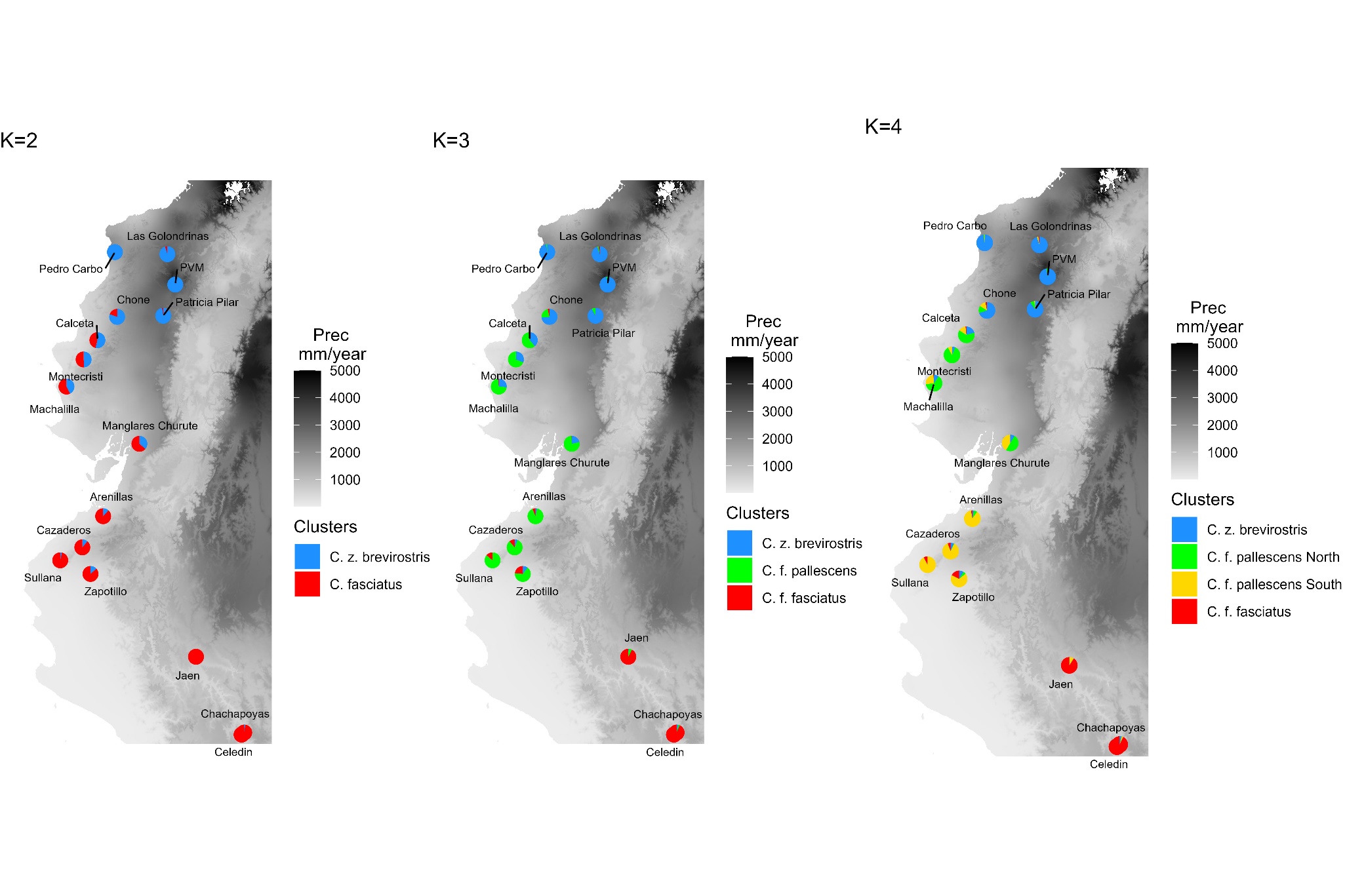


Figure S4. Genetic structure of *Campylorhynchus zonatus brevirostris* and *C. fasciatus* across an environmental gradient in western Ecuador and northern Peru. Pie charts illustrate mean individual assignment probabilities (Q) to K = 2-4 genetic clusters from a Bayesian analysis of ddRADseq data in STRUCTURE. At K = 3, C. fasciatus separates into the subspecies *C. f. pallescens* and *C. f. fasciatus*, with *pallescens* further divided into northern and southern clusters at K = 4. Slice widths correspond to locality mean Q. Background shading represent annual precipitation from WorldClim (~1 km2 resolution).


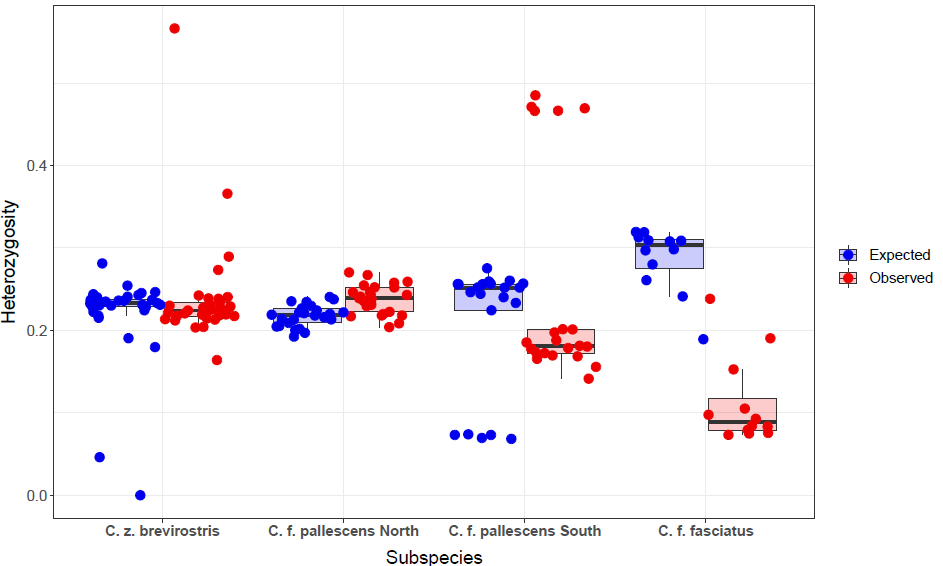


Figure S5. Genetic diversity in *Campylorhynchus zonatus brevirostris* and *C. fasciatus* subspecies/genetic clusters. Bars represent median observed and expected heterozygosity estimates derived from ddRADseq data, with 95% confidence intervals indicated. Genetic clusters within *C. fasciatus* were delineated by Bayesian analysis into the northern *pallescens* group, southern *pallescens* group, and nominate fasciatus subspecies. Higher observed versus expected heterozygosity in the northern *pallescens* cluster suggests elevated diversity from admixture with *C. z. brevirostris* compared to the other taxa.


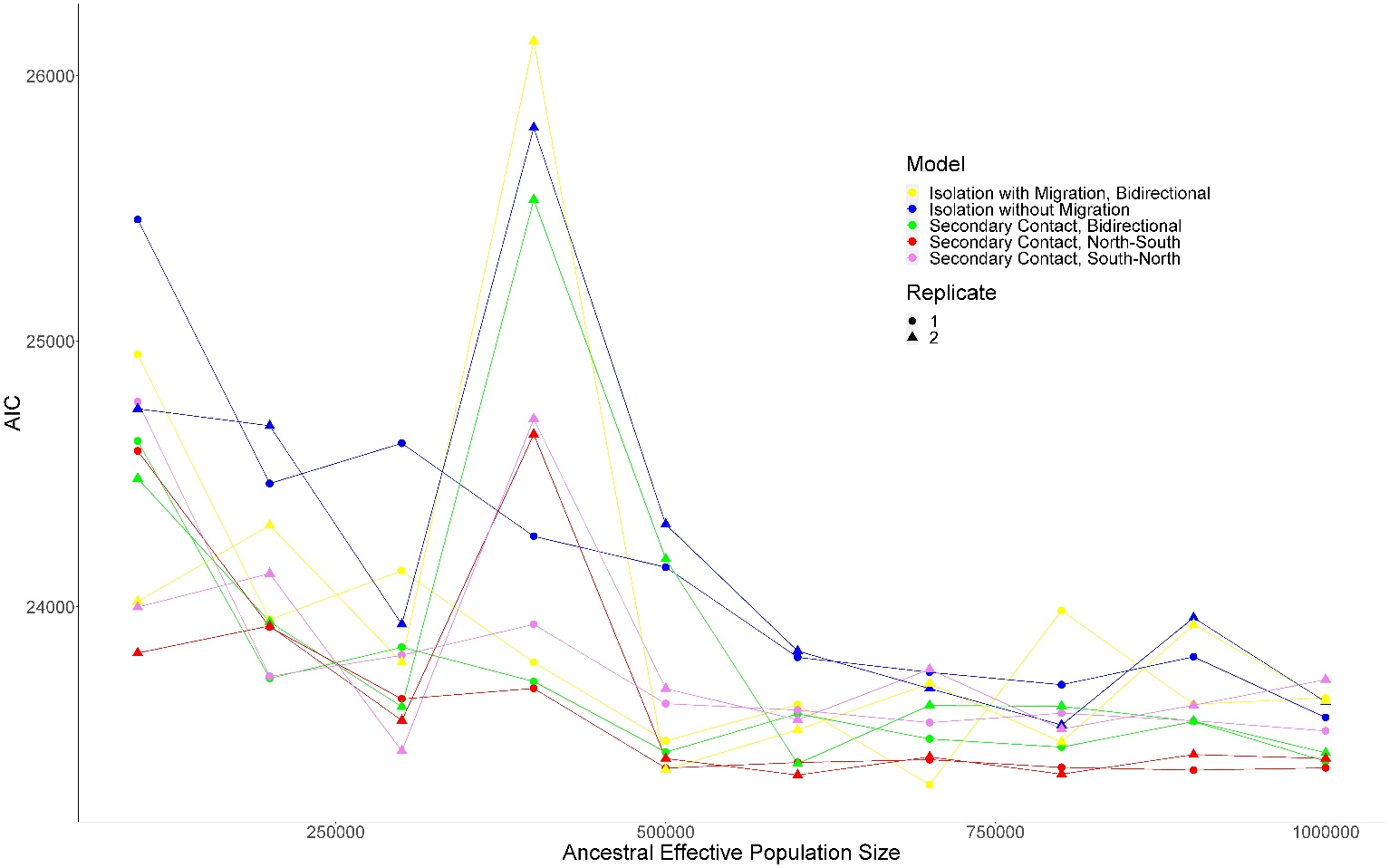
Figure S6. Influence of varying ancestral effective population sizes (Ne) on the Akaike Information Criterion (AIC) scores for five demographic models in *Campylorhynchus* species. The analysis utilizes ddRADseq data from *Campylorhynchus zonatus brevirostris* and *C. fasciatus pallescens* collected in western Ecuador across a climatic transition zone, implemented within the Momi2 framework. Among the models tested, the Isolation with Secondary Contact scenario and gene flow from the former to the latter species consistently yielded the lowest AIC values (approximately 23,800.64 in both replicates) when the ancestral Ne was around ${4.7\times10}^{5}$, suggesting this is the model that best represents the demographic history of these populations.

Table S1. Metadata on the individuals of *Campylorhynchus zonatus brevirostris* and *C. fasciatus* sampled from western Ecuador and northern Peru. It includes details regarding the sampling locality, designated breeding group, and corresponding geographic coordinates (latitude and longitude) for each individual.

| Sample | Locality | Breeding Group Code | Source | Museum code | Species | Lon | Lat |
| --- | --- | --- | --- | --- | --- | --- | --- |
| 1 | PVM | CZ020 | Field | NA | *C. zonatus* | -79.0504 | 0.07742 |
| 10 | Pedro Carbo | CZ029 | Field | NA | *C. zonatus* | -79.9315 | 0.39172 |
| 11 | Pedro Carbo | CZ034 | Field | NA | *C. zonatus* | -79.95 | 0.38649 |
| 12 | Pedro Carbo | CZ034 | Field | NA | *C. zonatus* | -79.95 | 0.38649 |
| 13 | Pedro Carbo | CZ031 | Field | NA | *C. zonatus* | -80.0224 | 0.49859 |
| 14B | PVM | CZ036 | Field | NA | *C. zonatus* | -79.0493 | -0.32027 |
| 15 | PVM | CZ036 | Field | NA | *C. zonatus* | -79.0493 | -0.32027 |
| 16 | PVM | CZ042 | Field | NA | *C. zonatus* | -79.0753 | -0.2152 |
| 17 | Patricia Pilar | CZ045 | Field | NA | *C. zonatus* | -79.2341 | -0.52827 |
| 18 | Patricia Pilar | CZ044 | Field | NA | *C. zonatus* | -79.2351 | -0.56201 |
| 1835 | Las Golondrinas | | Field | NA | *C. zonatus* | -79.1708 | 0.616614 |
| 1836 | Las Golondrinas | | Field | NA | *C. zonatus* | -79.1708 | 0.616614 |
| 19 | Patricia Pilar | CZ044 | Field | NA | *C. zonatus* | -79.2351 | -0.56201 |
| 2 | PVM | CZ021 | Field | NA | *C. zonatus* | -79.0512 | 0.06474 |
| 20 | Patricia Pilar | CZ049 | Field | NA | *C. zonatus* | -79.2358 | -0.51839 |
| 21 | Patricia Pilar | CZ050 | Field | NA | *C. zonatus* | -79.2357 | -0.54604 |
| 22 | Patricia Pilar | CZ050 | Field | NA | *C. zonatus* | -79.2357 | -0.54604 |
| 23 | Las Golondrinas | CZ058 | Field | NA | *C. zonatus* | -79.1981 | 0.285558 |
| 24B | Las Golondrinas | CZ058 | Field | NA | *C. zonatus* | -79.1981 | 0.285558 |
| 25 | Las Golondrinas | CZ060 | Field | NA | *C. zonatus* | -79.1607 | 0.269784 |
| 26 | Las Golondrinas | CZ061 | Field | NA | *C. zonatus* | -79.1404 | 0.23387 |
| 27 | Chone | CZ065 | Field | NA | *C. zonatus* | -79.7955 | -0.44534 |
| 28 | Chone | CZ070 | Field | NA | *C. zonatus* | -79.9847 | -0.56048 |
| 29 | Chone | CZ073 | Field | NA | *C. zonatus* | -79.937 | -0.58862 |
| 3 | PVM | CZ022 | Field | NA | *C. zonatus* | -79.0499 | 0.05464 |
| 30 | Chone | CZ074 | Field | NA | *C. zonatus* | -79.9241 | -0.58578 |
| 31 | Chone | CZ076 | Field | NA | *C. zonatus* | -80.0071 | -0.62203 |
| 32 | Calceta | CZ004 | Field | NA | *C. zonatus* | -80.1474 | -0.8114 |
| 34 | Calceta | CZ006 | Field | NA | *C. zonatus* | -80.1567 | -0.79718 |
| 35 | Calceta | CZ011 | Field | NA | *C. zonatus* | -80.1625 | -0.89254 |
| 36 | Calceta | CZ009 | Field | NA | *C. zonatus* | -80.1347 | -0.88784 |
| 37 | Calceta | CZ009 | Field | NA | *C. zonatus* | -80.1347 | -0.88784 |
| 38 | Calceta | CZ016 | Field | NA | *C. zonatus* | -80.253 | -0.86246 |
| 39 | Calceta | CZ016 | Field | NA | *C. zonatus* | -80.253 | -0.86246 |
| 4 | PVM | CZ023 | Field | NA | *C. zonatus* | -79.0486 | 0.04053 |
| 40 | Calceta | CZ016 | Field | NA | *C. zonatus* | -80.253 | -0.86246 |
| 41B | Calceta | CF001 | Field | NA | *C. zonatus* | -80.2372 | -0.90155 |
| 42 | Calceta | CF001 | Field | NA | *C. zonatus* | -80.2372 | -0.90155 |
| 43 | Calceta | CF001 | Field | NA | *C. zonatus* | -80.2372 | -0.90155 |
| 44 | Calceta | CF001 | Field | NA | *C. zonatus* | -80.2372 | -0.90155 |
| 45 | Calceta | CF012 | Field | NA | *C. zonatus* | -80.2793 | -0.97676 |
| 46 | Calceta | CF012 | Field | NA | *C. zonatus* | -80.2793 | -0.97676 |
| 47B | Calceta | CF012 | Field | NA | *C. fasciatus* | -80.2793 | -0.97676 |
| 48 | Calceta | CF017 | Field | NA | *C. fasciatus* | -80.2712 | -1.00968 |
| 49 | Calceta | CF017 | Field | NA | *C. fasciatus* | -80.2712 | -1.00968 |
| 50 | Calceta | CF017 | Field | NA | *C. fasciatus* | -80.2712 | -1.00968 |
| 51B | Montecristi | CF018 | Field | NA | *C. fasciatus* | -80.4164 | -1.18426 |
| 52B | Montecristi | CF018 | Field | NA | *C. fasciatus* | -80.4164 | -1.18426 |
| 53 | Montecristi | CF019 | Field | NA | *C. fasciatus* | -80.4197 | -1.21549 |
| 54 | Montecristi | CF019 | Field | NA | *C. fasciatus* | -80.4197 | -1.21549 |
| 55 | Montecristi | CF019 | Field | NA | *C. fasciatus* | -80.4197 | -1.21549 |
| 56 | Montecristi | CF019 | Field | NA | *C. fasciatus* | -80.4197 | -1.21549 |
| 57B | Montecristi | CF010 | Field | NA | *C. fasciatus* | -80.5349 | -1.18955 |
| 58B | Machalilla | CF034 | Field | NA | *C. fasciatus* | -80.7268 | -1.53592 |
| 59 | Machalilla | CF034 | Field | NA | *C. fasciatus* | -80.7268 | -1.53592 |
| 5B | PVM | CZ023 | Field | NA | *C. zonatus* | -79.0486 | 0.04053 |
| 6 | PVM | CZ024 | Field | NA | *C. zonatus* | -79.0576 | -0.05701 |
| 60 | Machalilla | CF035 | Field | NA | *C. fasciatus* | -80.7204 | -1.53714 |
| 61 | Machalilla | CF037 | Field | NA | *C. fasciatus* | -80.6832 | -1.51247 |
| 62 | Machalilla | CF039 | Field | NA | *C. fasciatus* | -80.666 | -1.67761 |
| 63 | Machalilla | CF040 | Field | NA | *C. fasciatus* | -80.6632 | -1.67787 |
| 64 | Machalilla | CF041 | Field | NA | *C. fasciatus* | -80.6849 | -1.67466 |
| 65B | Machalilla | CF041 | Field | NA | *C. fasciatus* | -80.6849 | -1.67466 |
| 66 | Machalilla | CF041 | Field | NA | *C. fasciatus* | -80.6849 | -1.67466 |
| 67B | Manglares Churute | CF043 | Field | NA | *C. fasciatus* | -79.6276 | -2.45463 |
| 68 | Manglares Churute | CF044 | Field | NA | *C. fasciatus* | -79.6286 | -2.42318 |
| 69 | Manglares Churute | CF050 | Field | NA | *C. fasciatus* | -79.528 | -2.54612 |
| 7 | PVM | CZ025 | Field | NA | *C. zonatus* | -79.0451 | -0.06608 |
| 71 | Arenillas | CF052 | Field | NA | *C. fasciatus* | -80.1426 | -3.56671 |
| 72 | Arenillas | CF052 | Field | NA | *C. fasciatus* | -80.1426 | -3.56671 |
| 73 | Arenillas | CF052 | Field | NA | *C. fasciatus* | -80.1426 | -3.56671 |
| 75B | Arenillas | CF053 | Field | NA | *C. fasciatus* | -80.1396 | -3.56699 |
| 76B | Arenillas | CF053 | Field | NA | *C. fasciatus* | -80.1396 | -3.56699 |
| 77B | Arenillas | CF053 | Field | NA | *C. fasciatus* | -80.1396 | -3.56699 |
| 79 | Arenillas | CF054 | Field | NA | *C. fasciatus* | -80.139 | -3.56193 |
| 80B | Zapotillo | CF062 | Field | NA | *C. fasciatus* | -80.2484 | -4.3889 |
| 82 | Zapotillo | CF063 | Field | NA | *C. fasciatus* | -80.2775 | -4.40015 |
| 83B | Zapotillo | CF063 | Field | NA | *C. fasciatus* | -80.2775 | -4.40015 |
| 85B | Zapotillo | CF065 | Field | NA | *C. fasciatus* | -80.3951 | -4.47825 |
| 86 | Zapotillo | CF065 | Field | NA | *C. fasciatus* | -80.3951 | -4.47825 |
| 88 | Zapotillo | CF066 | Field | NA | *C. fasciatus* | -80.3955 | -4.47422 |
| 8B | PVM | CZ026 | Field | NA | *C. zonatus* | -79.0628 | -0.09263 |
| 9 | PVM | CZ026 | Field | NA | *C. zonatus* | -79.0628 | -0.09263 |
| 92 | Cazaderos | CF076 | Field | NA | *C. fasciatus* | -80.4547 | -4.03596 |
| 93 | Cazaderos | CF076 | Field | NA | *C. fasciatus* | -80.4547 | -4.03596 |
| 94 | Cazaderos | CF076 | Field | NA | *C. fasciatus* | -80.4547 | -4.03596 |
| 95 | Cazaderos | CF076 | Field | NA | *C. fasciatus* | -80.4547 | -4.03596 |
| F001 | Jaen |  | FMNH | 50579 | *C. zonatus* | -78.7083 | -5.687 |
| F002 | Celedin |  | FMNH | 47494 | *C. zonatus* | -78.0233 | -6.85333 |
| F003 | Sullana |  | FMNH | 50573 | *C. zonatus* | -80.8405 | -4.22217 |
| F004 | Chachapoyas | | FMNH | 47591 | *C. zonatus* | -78.0152 | -6.82783 |
| F005 | Sullana |  | FMNH | 50574 | *C. zonatus* | -80.745 | -4.23883 |
| F006 | Sullana |  | FMNH | 50571 | *C. zonatus* | -80.745 | -4.23883 |
| F007 | Sullana |  | FMNH | 49499 | *C. zonatus* | -80.745 | -4.23883 |
| F008 | Chachapoyas | | FMNH | 47481 | *C. zonatus* | -78.0152 | -6.82783 |
| F009 | Jaen |  | FMNH | 49213 | *C. zonatus* | -78.7675 | -5.68383 |
| F010 | Celedin |  | FMNH | 47611 | *C. zonatus* | -78.0872 | -6.86567 |
| F011 | Jaen |  | FMNH | 49119 | *C. zonatus* | -78.7675 | -5.68383 |
| F012 | Sullana |  | FMNH | 50570 | *C. zonatus* | -80.745 | -4.23883 |
| F013 | Chachapoyas | | FMNH | 47685 | *C. zonatus* | -78.0152 | -6.82783 |
| F014 | Jaen |  | FMNH | 49281 | *C. zonatus* | -78.7675 | -5.68383 |
| F015 | Jaen |  | FMNH | 49189 | *C. zonatus* | -78.7675 | -5.68383 |
| F016 | Chachapoyas | | FMNH | 47571 | *C. zonatus* | -78.0152 | -6.82783 |
| F017 | Sullana |  | FMNH | 49416 | *C. zonatus* | -80.745 | -4.23883 |
| F019 | Sullana |  | FMNH | 49442 | *C. zonatus* | -80.8405 | -4.22217 |
| F020 | Jaen |  | FMNH | 50578 | *C. zonatus* | -78.7083 | -5.687 |
| F021 | Sullana |  | FMNH | 49368 | *C. zonatus* | -80.8405 | -4.22217 |
| F022 | Chachapoyas | | FMNH | 47420 | *C. zonatus* | -78.0152 | -6.82783 |
| F023 | Jaen |  | FMNH | 50577 | *C. zonatus* | -78.7083 | -5.687 |
| F024 | Sullana |  | FMNH | 49313 | *C. fasciatus* | -80.8405 | -4.22217 |
| F025 | Celedin |  | FMNH | 47457 | *C. fasciatus* | -78.0872 | -6.86567 |
| F026 | Celedin |  | FMNH | 47664 | *C. fasciatus* | -78.0233 | -6.85333 |

Table S2. Summary of sample size per sampling location, centroid of the coordinates, species and subspecies of *Campylorhynchus zonatus brevirostris* and *C. fasciatus* along western Ecuador and northern Peru, and the weather the samples came from the Florida Museum of Natural History (FMNH) or the field work of this study.

| Sampling Location | Lat | Lon | Sample Size | Species/Subspecies | Source |
| --- | --- | --- | --- | --- | --- |
| Arenillas | -3.566 | -80.141 | 7 | *C. fasciatus pallescens* | Field |
| Calceta | -0.913 | -80.228 | 18 | *C. zonatus brevirostris* | Field |
| Cazaderos | -4.036 | -80.455 | 4 | *C. fasciatus pallescens* | Field |
| Celedin | -6.860 | -78.055 | 4 | *C. fasciatus fasciatus* | FMNH |
| Chachapoyas | -6.828 | -78.015 | 5 | *C. fasciatus fasciatus* | FMNH |
| Chone | -0.560 | -79.930 | 5 | *C. zonatus brevirostris* | Field |
| Jaen | -5.685 | -78.742 | 7 | *C. fasciatus fasciatus* | FMNH |
| Las Golondrinas | 0.385 | -79.173 | 6 | *C. zonatus brevirostris* | Field |
| Machalilla | -1.611 | -80.693 | 9 | *C. fasciatus pallescens* | Field |
| Manglares Churute | -2.475 | -79.595 | 3 | *C. fasciatus pallescens* | Field |
| Montecristi | -1.203 | -80.435 | 7 | *C. fasciatus pallescens* | Field |
| Patricia Pilar | -0.544 | -79.235 | 6 | *C. zonatus brevirostris* | Field |
| Pedro Carbo | 0.416 | -79.963 | 4 | *C. zonatus brevirostris* | Field |
| Pedro Vicente Maldonado | -0.074 | -79.054 | 12 | *C. zonatus brevirostris* | Field |
| Sullana | -4.231 | -80.787 | 9 | *C. fasciatus pallescens* | FMNH |
| Zapotillo | -4.437 | -80.332 | 6 | *C. fasciatus pallescens* | Field |

Table S3. Summary of the genetic statistics for genetic clusters of *Campylorhynchus zonatus* and *C. fasciatus* populations from western Ecuador and northern Peru, as identified by the STRUCTURE software analysis at K = 4 based on ddRADseq data and de novo assembly. For each cluster, the following information is provided: N is the number of individuals, nLoc is the total number of loci analyzed, polyLoc is the number of polymorphic loci, and monoLoc is the number of monomorphic loci. Percentiles (2.5th, 25th, 50th, 75th, and 97.5th) of observed heterozygosity (Ho) and expected heterozygosity (He) are reported across individuals within each cluster. The inbreeding coefficient (Fis) percentiles summarize the deficiency or excess of heterozygotes relative to Hardy-Weinberg expectations.

| Genetic Clusters | N | nLoc | polyLoc | monoLoc | Ho Percentiles | | | | | He Percentiles | | | | | Fis |
| --- | --- | --- | --- | --- | --- | --- | --- | --- | --- | --- | --- | --- | --- | --- | --- |
|  |  |  |  |  | 2.5th | 25th | 50th | 75th | 97.5th | 2.5th | 25th | 50th | 75th | 97.5th |  |
| C. z. brevirostris | 24 | 4409 | 4254 | 155 | 0.20 | 0.22 | 0.22 | 0.23 | 0.41 | 0.04 | 0.23 | 0.23 | 0.24 | 0.26 | 0.21 |
| C. f. pallescens North | 26 | 4409 | 4322 | 87 | 0.21 | 0.22 | 0.24 | 0.25 | 0.27 | 0.20 | 0.21 | 0.22 | 0.23 | 0.24 | 0.20 |
| C. f. pallescens South | 24 | 4409 | 4341 | 68 | 0.15 | 0.17 | 0.18 | 0.20 | 0.48 | 0.07 | 0.22 | 0.25 | 0.26 | 0.27 | 0.23 |
| C. f. fasciatus | 12 | 4409 | 3918 | 491 | 0.07 | 0.08 | 0.09 | 0.12 | 0.23 | 0.20 | 0.28 | 0.30 | 0.31 | 0.32 | 0.23 |

Table S4. Analysis of Molecular Variance (AMOVA) for *Campylorhynchus zonatus brevirostris* and *Campylorhynchus fasciatus*, with the genetic clusters assigned by the Structure software for K=4. It shows the sum of squares (SSD), mean square deviation (MSD), degrees of freedom (df), variance components (sigma2), the percentage of variation among and within clusters, and the p-value indicating the statistical significance of the observed variation. The results demonstrate a significant 14.596% variation among genetic clusters, with the remainder attributed to within-cluster variation, indicating a structured genetic population.

|  | SSD | MSD | df | sigma2 | Percentage Variation | P.value |
| --- | --- | --- | --- | --- | --- | --- |
| Among Genetic Clusters | 0.357 | 0.119 | 3.000 | 0.004 | 14.596 | 0.0003 |
| Error | 2.184 | 0.025 | 87.000 | 0.025 | 85.404 |  |
| Total | 2.541 | 0.028 | 90.000 |  |  |  |

Table S5. Pairwise Nei's Fst estimates between *Campylorhynchus zonatus brevirostris* and *Campylorhynchus fasciatus* genetic clusters based on ddRADseq data analyzed with a de novo assembly pipeline. The *C. fasciatus* clusters were defined as the southern *pallescens* group, northern *pallescens* group, and nominate fasciatus subspecies from a Bayesian analysis in STRUCTURE at K = 4 genetic clusters. Higher Fst values indicate greater genetic divergence and lower inter-taxon gene flow.

| Genetic Clusters | *C. z. brevirostris* | *C. f. pallescens South* | *C. f. pallescens North* | *C. f. fasciatus* |
| --- | --- | --- | --- | --- |
| *C. z. brevirostris* |  | 0.088 | 0.078 | 0.058 |
| *C. f. pallescens* South | 0.088 |  | -0.006 | 0.000 |
| *C. f. pallescens* North | 0.078 | -0.006 |  | 0.002 |
| *C. f. fasciatus* | 0.058 | 0.000 | 0.002 |  |

Table S6. Climatic variables at sampling locations for *Campylorhynchus zonatus brevirostris* and *C. fasciatus pallescens/fasciatus* genetic clusters. Average monthly temperature (AMT, °C), annual mean precipitation (AMP, mm/year), and precipitation seasonality (PS, coefficient of variation) are shown for each sampling location within the ranges of the respective genetic clusters in western Ecuador and northern Peru. Values represent means with 95% confidence intervals in parentheses. Climate variables come from CHELSA 1.2 (Karger et al., 2017).

| Genetic Cluster | Sampling Locations | AMT (95% CI) | AMP (95% CI) | PS (95% CI) |
| --- | --- | --- | --- | --- |
| *C. z. brevirostris* | Las Golondrinas | 23.967 (23.651-24.282) | 2717.167 (2545.088-2889.249) | 57.333 (55.235-59.432) |
| *C. z. brevirostris* | PVM | 22.400 (21.839-22.961) | 2661.833 (2486.767-2836.900) | 62.917 (60.746-65.087) |
| *C. z. brevirostris* | Patricia Pilar | 23.783 (23.711-23.856) | 2058.167 (1995.321-2121.012) | 75.167 (74.914-75.420) |
| *C. z. brevirostris* | Chone | 23.200 (22.803-23.597) | 1476.200 (1405.542-1546.858) | 98.400 (96.725-100.075) |
| *C. z. brevirostris* | Pedro Carbo | 24.500 (24.428-24.571) | 1227.500 (1152.416-1302.584) | 84.000 (83.494-84.506) |
| *C. f. pallescens* North | Calceta | 23.617 (23.261-23.973) | 785.111 (701.154-869.069) | 106.389 (105.562-107.216) |
| *C. f. pallescens* North | Montecristi | 23.343 (22.921-23.765) | 557.429 (533.509-581.348) | 107.143 (106.909-107.377) |
| *C. f. pallescens* North | Machalilla | 22.689 (22.556-22.822) | 545.778 (524.109-567.446) | 110.222 (108.953-111.492) |
| *C. f. pallescens* North | Manglares Churute | 25.800 (25.696-25.907) | 990.000 (920.767-1059.233) | 103.000 (100.765-105.235) |
| *C. f. pallescens* South | Arenillas | 25.014 (24.991-25.038) | 644.000 (641.681-646.319) | 101.857 (101.623-102.091) |
| *C. f. pallescens* South | Cazaderos | 23.900 (23.900-23.900) | 353.000 (353.000-353.000) | 127.000 (127.000-127.000) |
| *C. f. pallescens* South | Zapotillo | 25.217 (25.170-25.263) | 287.667 (251.863-323.471) | 149.500 (146.849-152.151) |
| *C. f. pallescens* South | Sullana | 21.022 (19.977-22.068) | 228.333 (194.033-262.633) | 144.889 (141.296-148.482) |
| *C. f. fasciatus* | Jaen | 24.514 (24.117-24.912) | 893.000 (890.681-895.319) | 32.571 (32.240-32.903) |
| *C. f. fasciatus* | Celedin | 20.550 (18.510-22.590) | 797.500 (666.886-928.114) | 47.500 (47.142-47.858) |
| *C. f. fasciatus* | Chachapoyas | 23.600 (23.600-23.600) | 564.000 (564.000-564.000) | 46.000 (46.000-46.000) |

Table S7. Parameter estimates obtained from Momi2 analyses testing various demographic models of divergence and gene flow between *Campylorhynchus zonatus brevirostris* (CZ) and *C. fasciatus pallescens* (CFP) based on ddRADseq data. Summary statistics are provided for key parameters under each model, including effective population sizes at time of divergence (Necfp_tdiv, Necz_tdiv), time of divergence in years (tdiv), migration rates (msn = from CZ to CFP; mns = from CFP to CZ), and timing of migration pulses (tmigsn, tmigns). The table reports the median, mean, and 95% confidence intervals for each parameter under the different demographic scenarios: isolation without migration, isolation with symmetric migration, secondary contact, asymmetric migration from south to north (CFP->CZ), and asymmetric migration from north to south (CZ->CFP). The best supported model (*) was isolation with asymmetric gene flow from CZ into CFP.

| Demographic Scenario | Migration Direction | Parameter | Median | Mean | Lower 95% CI | Upper 95% CI |
| --- | --- | --- | --- | --- | --- | --- |
| Isolation without Migration | No Migration | Necfp_tdiv | 8.00E+04 | 9.97E+04 | 7.86E+04 | 1.21E+05 |
|  |  | Necz_tdiv | 1.78E+05 | 2.47E+05 | 1.79E+05 | 3.14E+05 |
|  |  | tdiv | 9.69E+04 | 1.49E+05 | 1.06E+05 | 1.91E+05 |
| Isolation with Migration | Bidirectional CFP<->CZ | Necfp_tdiv | 6.97E+04 | 5.66E+04 | 5.19E+04 | 6.12E+04 |
|  |  | Necz_tdiv | 2.78E+06 | 2.78E+06 | 2.78E+06 | 2.79E+06 |
|  |  | msn | 30.40% | 41.95% | 32.34% | 51.55% |
|  |  | mns | 38.74% | 42.48% | 34.23% | 50.73% |
|  |  | tdiv | 9.26E+04 | 9.26E+04 | 8.48E+04 | 1.00E+05 |
| Secondary Contact | Bidirectional CFP<->CZ | Necfp_tdiv | 5.78E+04 | 5.70E+04 | 5.17E+04 | 6.22E+04 |
|  |  | Necz_tdiv | 2.77E+06 | 2.78E+06 | 2.77E+06 | 2.79E+06 |
|  |  | msn | 27.42% | 35.19% | 26.70% | 43.68% |
|  |  | mns | 27.93% | 36.70% | 28.78% | 44.62% |
|  |  | tmigsn | 1.15E+04 | 1.03E+04 | 8.95E+03 | 1.16E+04 |
|  |  | tmigns | 1.21E+04 | 1.08E+04 | 9.70E+03 | 1.20E+04 |
|  |  | tdiv | 9.26E+04 | 9.26E+04 | 8.48E+04 | 1.00E+05 |
|  | North-South* CZ->CFP | Necfp_tdiv | 6.87E+04 | 6.43E+04 | 5.90E+04 | 6.96E+04 |
|  |  | Necz_tdiv | 2.77E+06 | 2.78E+06 | 2.77E+06 | 2.79E+06 |
|  |  | mns | 33.38% | 37.11% | 30.01% | 44.21% |
|  |  | tmigns | 9.61E+03 | 9.41E+03 | 8.13E+03 | 1.07E+04 |
|  |  | tdiv | 9.26E+04 | 9.26E+04 | 8.48E+04 | 1.00E+05 |
|  | South-North CFP->CZ | Necfp_tdiv | 9.06E+04 | 9.59E+04 | 8.31E+04 | 1.09E+05 |
|  |  | Necz_tdiv | 2.77E+06 | 2.77E+06 | 2.77E+06 | 2.78E+06 |
|  |  | msn | 25.94% | 33.75% | 25.52% | 41.99% |
|  |  | tmigsn | 1.15E+04 | 1.05E+04 | 9.26E+03 | 1.18E+04 |
|  |  | tdiv | 9.26E+04 | 9.26E+04 | 8.48E+04 | 1.00E+05 |
